# Global cortical arousal effects in fMRI reveal brain markers of state and trait anxiety

**DOI:** 10.1093/cercor/bhag008

**Published:** 2026-02-17

**Authors:** Kimberly Kundert-Obando, Terra Lee, Caroline G Martin, Kamalpreet Kaur, Juan Gomez Lagandara, Yamin Li, Jeffrey M Harding, Shiyu Wang, Richard Song, Ruoqi Yang, Rithwik Guntaka, Sarah E Goodale, Roza G Bayrak, Lucina Q Uddin, Martin Walter, Jeremy Hogeveen, Catie Chang

**Affiliations:** Vanderbilt Neuroscience Graduate Program, Vanderbilt University, 465 21st Ave S, Nashville, TN 37232, United States; Vanderbilt Brain Institute, 265 21st Avenue South, Nashville, TN 37240, United States; Vanderbilt University Medical Center, 1211 Medical Center Dr, Nashville, TN 37232, United States; Department of Psychology and Human Development, Peabody College, Vanderbilt University, 1210 21st Ave S, Nashville, TN 37212, United States; Program in Neuroscience, Vanderbilt University, 2305 West End Ave, Nashville, TN 37203, United States; Vanderbilt University Medical Center, 1211 Medical Center Dr, Nashville, TN 37232, United States; Department of Electrical and Computer Engineering, Vanderbilt University, 2301 Vanderbilt Place, Nashville, TN 37235-1824, United States; Department of Electrical and Computer Engineering, Vanderbilt University, 2301 Vanderbilt Place, Nashville, TN 37235-1824, United States; Department of Electrical and Computer Engineering, Vanderbilt University, 2301 Vanderbilt Place, Nashville, TN 37235-1824, United States; Department of Computer Science, Vanderbilt University, Sony Big, 1400 18th Ave S, Nashville, TN 37212, United States; Department of Electrical and Computer Engineering, Vanderbilt University, 2301 Vanderbilt Place, Nashville, TN 37235-1824, United States; Department of Biomedical Engineering, Vanderbilt University, Stevenson Center Ln, Nashville, TN 37240, United States; Program in Neuroscience, Vanderbilt University, 2305 West End Ave, Nashville, TN 37203, United States; Department of Computer Science, Vanderbilt University, Sony Big, 1400 18th Ave S, Nashville, TN 37212, United States; Department of Computer Science, Vanderbilt University, Sony Big, 1400 18th Ave S, Nashville, TN 37212, United States; Department of Computer Science and Engineering, University of California San Diego, 9500 Gilman Dr, La Jolla, CA 92093, United States; Department of Electrical and Computer Engineering, Vanderbilt University, 2301 Vanderbilt Place, Nashville, TN 37235-1824, United States; Department of Biomedical Engineering, Vanderbilt University, Stevenson Center Ln, Nashville, TN 37240, United States; Vanderbilt Memory and Alzheimer's Center, Vanderbilt University Medical Center, 1211 Medical Center Drive, Nashville, TN 37232, United States; Department of Electrical and Computer Engineering, Vanderbilt University, 2301 Vanderbilt Place, Nashville, TN 37235-1824, United States; Department of Psychiatry and Biobehavioral Sciences, University of California Los Angeles, 744 Westwood Plaza, Los Angeles, CA 90095, United States; Department of Psychology, University of California Los Angeles, 405 Hilgard Avenue, Los Angeles, CA 90095-1563, United States; University Clinic for Psychiatry and Psychotherapy, University Hospital Jena, Am Steiger 3, 07743 Jena, Germany; Department of Psychology, University of New Mexico, 1820 Sigma Chi Rd NE, Albuquerque, NM 87106, United States; Vanderbilt Brain Institute, 265 21st Avenue South, Nashville, TN 37240, United States; Department of Electrical and Computer Engineering, Vanderbilt University, 2301 Vanderbilt Place, Nashville, TN 37235-1824, United States; Department of Computer Science, Vanderbilt University, Sony Big, 1400 18th Ave S, Nashville, TN 37212, United States; Department of Biomedical Engineering, Vanderbilt University, Stevenson Center Ln, Nashville, TN 37240, United States

**Keywords:** arousal, fmri, global mean signal, state anxiety, trait anxiety

## Abstract

To personalize the diagnosis and treatment of anxiety, there is a need to identify biological constructs that underlie self-reported symptoms. Notably, physiological responses and levels of arousal are constituents of anxiety, and have widespread (“global”) effects on functional magnetic resonance imaging (fMRI) signals that may provide novel brain markers of anxiety. Here, we modeled autonomic physiological and cortical arousal signatures in fMRI data and determined whether these global fMRI components relate to measures of state and trait anxiety. Additionally, we tested if these global effects impact relationships between brain network connectivity and anxiety levels. We found significant relationships between fMRI global components and state/trait anxiety scores, identifying brain regions in which the strength of the global mean fMRI signal and the cortical arousal-related fMRI signal were associated with both state and trait anxiety. Notably, the resulting patterns exhibited substantial overlap with established large-scale functional networks, including the default-mode network. These findings indicate that global effects in fMRI signals hold valuable information that may reveal how cortical arousal is expressed in anxiety, and may provide a source of information that has previously been treated as a confound.

## Introduction

In 2019, approximately 301 million individuals worldwide were diagnosed with an anxiety disorder (Alonso et al. 2018; Global Burden of Disease Collaborative Network 2020). The current practice for diagnosing an anxiety disorder relies on the Diagnostic and Statistical Manual of Mental Disorders (Harm et al. 2013), which is based on an individual’s behavior. Yet, behavioral assessments can be subjective, leading to inconsistent and/or inaccurate diagnoses. To address this limitation, the National Institute of Mental Health initiated the Research Domain Criteria (RDoC) to integrate multiple levels of information from neural circuits to behavior to better understand mental health and illness.

A widespread approach for investigating biomarkers of anxiety centers on the functional connectivity of large-scale brain networks measured with functional magnetic resonance imaging (fMRI). Overall, large-scale functional network connectivity tends to show a weak association with anxiety (Xu et al. 2019). Nonetheless, multiple studies have reported that the functional connectivity within and between two major brain networks—the salience network (Uddin 2016, 2015) and the default-mode network (Mason et al. 2007)—relates to inter-individual differences in state and trait anxiety (Dennis et al. 2011; Modi et al. 2015; Saviola et al. 2020). However, the precise relationship between brain network functional connectivity and anxiety appears to be complex. For instance, the inter- and intra-network connectivity of the salience network and the default mode network has been observed in some studies to increase (Sylvester et al. 2012; Burdwood et al. 2016) but in others to decrease (Sylvester et al. 2012; Modi et al. 2015; Geng et al. 2016) as anxiety increases. These inconsistencies motivate a continued search for more robust biological markers of anxiety.

In separate lines of work, anxiety has been closely linked with measures of autonomic physiology (eg heart rate) (Dimitriev et al. 2016; Williams et al. 2017; Tomasi et al. 2024) and cortical arousal (Valle and DeGood 1977). However, these processes have been under-explored in the context of neuroimaging biomarkers of anxiety. Fluctuations in heart rate (Shmueli et al. 2007; Chang and Glover 2009; Pfurtscheller et al. 2021) and cortical arousal (Falahpour et al. 2018; Liu and Falahpour 2020; Martin et al. 2021) are known to manifest in fMRI signals, exhibiting widespread (“global”) effects and propagating patterns (Bolt et al. 2024; Gu et al. 2022; Mäki-Marttunen and Nieuwenhuis 2025; Raut et al. 2025, 2021). It is therefore possible that these effects may help to identify stronger biomarkers of anxiety and reveal brain regions that relate physiological or cortical arousal to anxiety.

While fMRI global components are often considered to be artifactual, growing evidence suggests that they may hold valuable information related to anxiety (Gu et al. 2020, Gaebler et al. 2013; Li et al. 2019b, 2019a; Long et al. 2024; Mujica-Parodi et al. 2007; Pfurtscheller et al. 2021). For example, studies measuring heart rate variability during fMRI have identified brain-physiological interactions in several areas that are related to anxiety (Mujica-Parodi et al. 2007; Gaebler et al. 2013; Pfurtscheller et al. 2021). The average fMRI signal across the brain (“global mean signal”), which includes both arousal and heart rate effects, was found to be expressed in brain regions closely tied with gene expression and neurotransmitters crucial to trait anxiety (Long et al. 2024). To date, fMRI studies of cortical arousal have been limited, as validated arousal measures are not commonly acquired during fMRI. However, approaches for estimating fMRI cortical arousal components have been developed (Chang et al. 2016; Falahpour et al. 2018; Goodale et al. 2021). An fMRI spatial pattern that tracked arousal fluctuations measured by cortical local field potential recordings and eye closures was identified in macaques (Chang et al. 2016) and a similar pattern was subsequently identified in humans using a simultaneous fMRI and an EEG arousal measure (defined as the ratio of power in the beta band to that of delta and theta bands) (Falahpour et al. 2018). Goodale et al. replicated this fMRI cortical arousal pattern in an independent human dataset and also demonstrated that it could predict behavioral task performance (Goodale et al. 2021). This methodological advance can be leveraged to study cortical arousal in fMRI studies that do not acquire measures such as EEG or pupillometry during fMRI. Here we leveraged this novel technique to study the association between fMRI arousal and anxiety.

In the present study, we conducted an exploratory analysis using data from a large sample of individuals from the Enhanced Nathan Kline Institute-Rockland Sample (Nooner et al. 2012) to determine if global fMRI components may provide brain-based markers of state or trait anxiety. We focus on three global fMRI components: (1) a data-driven estimate of cortical arousal-dependent fMRI fluctuations that has been replicated across species and different sites (Chang et al. 2016; Falahpour et al. 2018; Goodale et al. 2021), (2) the global mean signal, and (3) fMRI correlates of heart rate. While the NKI data is not drawn from a clinical population, it provides a representative sample of anxiety in the general population. This analysis therefore provides foundational knowledge and methodology for future investigations in clinical populations.

In addition, while global components are often studied independently of functional networks, arousal and autonomic processes have been found to exhibit structured spatiotemporal relationships with functional networks (Chen et al. 2020; Raut et al. 2021; Raut et al. 2025). As such, the activity of a given network likely reflects a mixture of global (arousal, autonomic) and network-specific activity, and it is unclear which of these components is more closely linked with measures of anxiety. Therefore, we conducted secondary analyses to examine how the association between global components and anxiety aligns with major large-scale functional networks, and how removing global components impacts the relationship between network connectivity and anxiety. We focus specifically on the default mode, salience, and central executive networks, which comprise the triple-network model (Menon and Uddin 2010; Menon 2011), because their interactions have been shown to be sensitive to arousal (Young et al. 2017) and are also implicated in anxiety (Sylvester et al. 2012).

Taken together, these complementary analyses aim to (1) provide new insight into the value of brain-wide physiological and cortical arousal signatures as markers of anxiety, (2) clarify the extent to which information is shared across global components and large-scale functional networks in their association with anxiety, and (3) inform how interactions between major brain networks and anxiety are impacted by modeling global fMRI effects. Overall, the results of these analyses have implications for the construction of brain-based biomarkers and the interpretation of brain network-anxiety associations.

## Methods and materials

### Data acquisition

Data were drawn from the Enhanced Nathan Kline Institute-Rockland Sample (NKI-RS; (Nooner et al. 2012)). All procedures were performed in compliance with relevant laws and institutional guidelines and have been approved by the Institutional Review Boards of the NKI (Phase I #226781 and Phase II #239708) and Montclair State University (Phase I #000983A and Phase II #000983B). Written informed consent was obtained for all study participants. The neuroimaging data was retrieved at https://fcon_1000.projects.nitrc.org/indi/enhanced/, and phenotypic data was received after signing a data usage agreement. Resting-state fMRI (multiband EPI sequence, TR = 1400 ms, duration = 10 min, voxel size = 2.0 mm isotropic, FA = 65°, FOV = 224 mm) and a high-resolution anatomic scan (MPRAGE sequence, TR/TE = 1900/2.52 ms, FA = 9°, thickness = 1.0 mm, slices = 192, matrix = 256 × 256, FOV = 250 mm) were acquired for each participant. Cardiac (via photoplethysmography; PPG) data were recorded during the resting-state fMRI scan. A total of 543 subjects that had both fMRI and State–Trait Anxiety Inventory scores (F = 369, M = 174) were included. All 543 subjects completed the State–Trait Anxiety Inventory (STAI) approximately one day before the MRI scan. Our analyses used the T-scored values as obtained per the guidelines of the STAI scoring manual.

### MRI data preprocessing

The T1 anatomic images underwent brain extraction (FSL *bet*), reduction of spatial bias field inhomogeneities (AFNI *3dUnifize*), and a nonlinear transformation between the T1 image and MNI152 space (ANTS). The fMRI data first underwent motion coregistration (FSL *mcflirt*) followed by ICA FIX (Salimi-Khorshidi et al. 2014) to correct for motion and scanner artifacts. For FIX, we constructed a study-specific training set using 25 subjects from the NKI dataset with a balanced age distribution. The fMRI data were then registered to the associated T1 image with an affine transformation (FSL *epi_reg*), followed by alignment to MNI152 space via the previously estimated nonlinear transformation between T1 and MNI152 space. Spatial blurring was then performed (AFNI *3dmerge*; FWHM of 3 mm), and slow scanner drifts were reduced by removing polynomials up to a 4th order (AFNI *3dDetrend*).

### Heart rate preprocessing

To calculate HR, we first band-pass filtered the photoplethysmography data with a second-order Butterworth filter (0.5 to 2 Hz), detected peaks with a minimum height of 5% of the interquartile range, and calculated the time between successive peaks as the inter-beat interval (IBI). Although scans having high-quality PPG data had been selected for analysis, some of these nonetheless contained transient, fixable artifacts, and we interpolated over the few instances when these occurred. We then derived HR as the inverse of the median IBI per minute within successive 6-s sliding windows centered on the time of each fMRI volume acquisition (ie at each TR)(Chang et al. 2009; Chen et al. 2020). Two trained researchers manually inspected the raw PPG data and the derived HR measures to ensure the quality of the data. This yielded a set of 240 subjects with sufficient HR data quality, which were used in analyses that involved HR (Supplementary Fig. S1).

### Deriving global fMRI components

For each subject, we used a template-based approach for deriving arousal effects in fMRI (Chang et al. 2016; Falahpour et al. 2018; Goodale et al. 2021). Briefly, an fMRI Arousal Index (FAI) time-series was derived by first z-scoring each voxel’s fMRI time-series, and then spatially correlating the template map derived in Goodale et al. 2021 onto the fMRI volume at each TR. In addition to this FAI time series, which provides an estimate of arousal fluctuations across the scan, a voxel-wise spatial map—corresponding to the strength at which this FAI is expressed at each voxel—was derived by projecting (via linear regression) the FAI time series onto the time-series of each voxel and extracting the corresponding regression coefficient (beta) at each voxel.

The fMRI global mean signal (GS) was calculated as the average time-series across all voxels in the brain. A voxel-wise spatial map, corresponding to the strength at which the GS is expressed at each voxel, was derived by projecting (via linear regression) the GS onto the time-series of each voxel and extracting the corresponding regression coefficient (beta) at each voxel.

Since the mapping between heart rate (HR) and fMRI cannot be captured by a simple correlation (Chang et al. 2009), we used basis functions (Chen et al. 2020) to compute five HR regressors after removing polynomial trends up to a 4th order (matching the detrending step of the fMRI data). We computed a map capturing HR effects across the brain by calculating the percentage of temporal variance explained by these HR regressors in each voxel’s fMRI time-series.

### Relating spatial characteristics of global components to anxiety measures

To identify brain regions in which the expression of each global component is associated with anxiety measures, we used six general linear models (implemented in FSL *randomize*) to relate subject-specific GS maps, heart rate variation maps, and FAI maps to state and trait anxiety scores while covarying for age, gender, ethnicity. In subsequent tests, we examined the impact of including an additional covariate for head motion (mean framewise displacement; FD) at the subject level. Correction for multiple comparisons was carried out using Threshold Free Cluster Enhancement (TFCE) with 5000 permutations, and significance was assessed at corrected threshold of *P* < 0.05. To compare the spatial distributions of significant relationships between global components and anxiety across maps, we constructed a conjunction map after binarizing the individual TFCE maps at a threshold at *P* < 0.05. Significant brain regions were identified by manually inspecting clusters that corresponded with the Automated Anatomical Labeling 3 atlas (Rolls et al. 2020). Since only the FAI and GS maps revealed significant clusters, we multiplied the binarized GS map by two and added the binarized FAI map. This resulted in a conjunction map containing the value of “3” in overlapping regions, “2” in regions that are significant only within the GS-anxiety map, and “1” in regions that are significant only within the FAI-anxiety map. Additionally, given the wide age range of the NKI sample, we probed the sensitivity of results to age by rerunning the analyses without age as a covariate, as well as by excluding participants older than 55 years while retaining age as a covariate (Nomi et al. 2024).

### Relating temporal characteristics of global components to anxiety measures

We also related temporal features of each global component to anxiety measures, including the standard deviation of the FAI time-series (which serves as an estimated drowsiness value) (Falahpour et al. 2018), the standard deviation of the GS (Wong et al. 2013), and the mean HR. We constructed six models that compared temporal features of the FAI, GS, and mean HR with state and trait anxiety, including age, sex, and ethnicity covariates in each model since these effects can significantly impact fMRI signals and heart rate (Lopez-Larson et al. 2011; Hutchison et al. 2013; Santos et al. 2013; Xin et al. 2019; Nomi et al. 2024). False discovery rate (FDR) at q = 0.05 correction for multiple comparisons across anxiety measures was subsequently carried out for each global component. We also tested the impact of covarying for mean framewise displacement at the group level. Since the NKI sample spanned a wide age range, we additionally assessed the robustness of the results to age by rerunning the analyses without age as a covariate, and by excluding participants older than 55 years while retaining age as a covariate (Nomi et al. 2024).

### Deriving resting-state functional networks

Group-level independent component analysis (ICA), implemented in FSL Melodic, was applied to derive resting-state functional networks from a randomly selected group of 50 subjects. To assess robustness to initial ICA dimensionality, we additionally ran Melodic with both 40 and 60 components. Since the components derived with these two dimensionalities were very similar, we used the 40-component ICA decomposition for our analysis. These components were visually inspected to identify five networks of interest: dorsal default mode network (DDMN), ventral default mode network (VDMN), salience network (SAL), left central executive network (LCEN), and right central executive network (RCEN). The ICA decomposition had split the DMN and CEN into two subnetworks each, and we maintained the results of this data-driven approach rather than combining the subnetworks.

### Spatial overlap between resting-state networks and the association between global components and anxiety

To understand how associations between anxiety and fMRI global components are spatially aligned with canonical large-scale functional networks, we calculated the percentage of significant voxels in the global component-anxiety spatial maps that overlapped with these networks. Specifically, we binarized each ICA component (melodic_IC.nii output) at a threshold of 1.17, which was empirically determined to retain the major nodes of each network, and multiplied the resulting maps with a binarized map of clusters (thresholded at a value of *P* < 0.05 corrected) in which the association between global components and both state and trait anxiety measures was significant. Finally, we calculated the proportion of each ICA network that overlapped with these significant clusters by dividing the number of overlapping voxels by the total number of voxels in the thresholded ICA network, providing an index of how much of each network was expressed within the univariate anxiety–global component maps.

### Relating global component signals to resting-state functional network signals

We conducted dual regression (Nickerson et al. 2017) with all 40 components to derive subject-specific time-series and spatial maps for each component. To understand the relationship between the time-series of the global components and those of the functional networks, we correlated the FAI and GS to each network of interest for the subset of 240 subjects with sufficient heart rate signal quality. For heart rate, we calculated 10 lagged cross-correlations and recorded the maximum correlation between heart rate and each network of interest. To test for differences in the strength of correlations between global components and networks, we conducted a two-way repeated ANOVA using Rstudio (Version 2024.12.0 + 467) for the subset of 240 subjects who had a measure for all three global components. For post hoc analyses, we controlled for multiple comparisons using FDR correction at q = 0.05, applied both within global components (across all tested network pairs) and between global components (for each network). We also tested whether network-component correlations are significantly different than 0 using a one-sample t-test with FDR correction at q = 0.05 for multiple comparisons across networks tested.

### Relating functional connectivity to state and trait anxiety measures

Pairwise functional connectivity was then computed amongst the five networks of interest, using Pearson correlation, both before and after regressing out each global component in turn from the network time-series. Next, we constructed a linear mixed-effects model that relates functional connectivity strength with state or trait anxiety score, covarying for age, sex, and ethnicity and correcting for FDR by at q = 0.05 for all tests. We replicated these procedures for the subset of 240 subjects with high-quality heart rate data. To test if associations between connectivity measures and anxiety were significantly impacted by regressing out a given global component, we constructed a linear mixed-effects model with an interaction term to test whether the slopes of the predictors (functional connectivity measures predicting anxiety measures) differed pre- and post-regression of global components while covarying for age, sex, and ethnicity and conducted FDR (q = 0.05) grouped by anxiety (state or trait) to control for multiple comparisons.

## Results

### Demographic results

Subject demographics are depicted in Table 1. From this cohort, 240 subjects (F = 154, M = 86) that had adequate HR quality were used in analyses that required heart rate. State and trait anxiety T-scores were significantly correlated (r = 0.75, *P* < 0.001).

**Table 1:** Demographic information and state/trait anxiety scores.

Characteristic	All subjects (*n* = 543)	Subjects with high-quality HR (*n* = 240)
Age	48.1 (±17.4)	43.8 (±16.7)
Sex	369/174 (F/M)	154/86 (F/M)
Ethnicity	480/63 (Not Hispanic, Latino, Spanish/Hispanic, Latino, Spanish)	206/34 (Not Hispanic, Latino, Spanish/Hispanic, Latino, Spanish)
State (t-scores)	46.8 (±10.2)	45.3 (±9.0)
Trait (t-scores)	50.7 (±11.6)	49.1 (±10.4)
Potential STAI Clinical Anxiety Population	97/446 (Clinical/Non-clinical)	32/208 (Clinical/Non-clinical)

Demographics and State and Trait Anxiety Inventory (STAI) t-scores for the indicated subsets of participants used in this study. Values in the table indicate mean (± standard deviation). STAI approximate Clinical Anxiety Ranges were calculated using the raw state. HR: heart rate.

### Spatial Associations Between Global fMRI Components and Anxiety Measures

**Fig 1:**
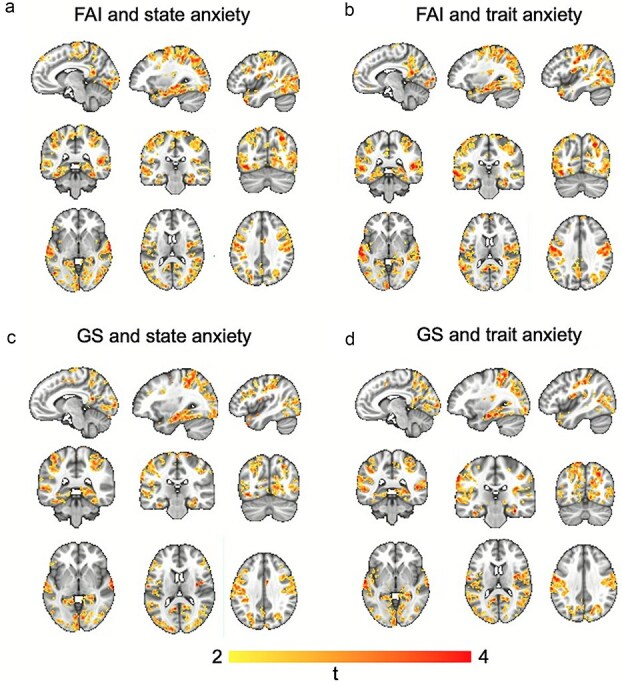
Spatial association between global components and anxiety. a and b) Areas in which the FAI was significantly associated with state and trait anxiety. c and d) Areas in which the GS was significantly associated with state and trait anxiety (GS was analyzed using a negative contrast). Maps show the t-statistics thresholded at *P* < 0.05 corrected.

The spatial maps of the FAI and of the GS exhibited significant relationships (*P* < 0.05, corrected) to state and trait anxiety (Fig. 1, Table 2). When covarying for mean FD at the group level, additional clusters emerged in the insula and posterior cingulate cortex (Supplementary Fig. S2). Significant clusters were present only in the positive contrast between FAI and anxiety, and only in the negative contrast between GS and anxiety, which can be attributed to the inverse relationship between FAI and GS (Supplementary Fig. S3). Therefore, the indicated clusters are those in which higher state or trait anxiety corresponded to a weaker regional amplitude of GS and FAI (specifically, weaker positive expression for GS, and weaker negative expression for FAI). No significant clusters were found for the heart-rate spatial maps; t-statistic maps are shown in Supplementary Fig. S4. When repeating the analysis with a subset of individuals between ages 18–55, only associations between state anxiety and FAI spatial maps showed significant clusters (Supplementary Fig. S5). Repeating the analysis with the full sample, but without covarying for age, revealed clusters similar to those obtained when covarying for age.

**Table 2:** Brain areas in which global fMRI components were associated with anxiety.

Global Component	State	Trait
FAI	Amygdala_R	Amygdala_R
	Angular_L	Angular (Bilateral)
	Calcarine_L	Calcarine_L
	Cerebelum_4_5_R	Cerebelum_6_R
	Cerebelum_6 (Bilateral)	Cerebelum_Crus1 (Bilateral)
	Cerebelum_Crus1 (Bilateral)	Cingulam_Ant_R
	Cingulam_Mid (Bilateral)	Cingulam_Mid_R
	Cingulam_Post (Bilateral)	Cingulam_Post_R
	Cuneus_R	Cuneus (Bilateral)
	Frontal_Inf_Oper (Bilateral)	Fusiform (Bilateral)
	Frontal_Inf_Tri_L	Frontal_Inf_Oper_L
	Frontal_Mid (Bilateral)	Frontal_Inf_Orb_L
	Fusiform (Bilateral)	Frontal_Inf_Tri_L
	Heschl_R	Frontal_Sup_Medial
	Hippocampus (Bilateral)	(Bilateral)
	Insula (Bilateral)	Fusiform (Bilateral)
	Lingual (Bilateral)	Heschl (Bilateral)
	Occipital_Inf_L	Hippocampus (Bilateral)
	Occipital_Mid_R	Insula (Bilateral)
	Occipital_Sup (Bilateral)	Lingual (Bilateral)
	Paracentral_Lobule	Occipital_Inf (Bilateral)
		Occipital_Mid_R
	(Bilateral) Parietal_Inf (Bilateral)	Occipital_Sup_L
	Parietal_Sup_L	Paracentral_Lobule_R
	ParaHippocampal_R	Parietal_Inf (Bilateral)
	Postcentral (Bilateral)	Parietal_Sup (Bilateral)
	Precentral (Bilateral)	ParaHippocampal (Bilateral)
	Precuneus (Bilateral)	Postcentral (Bilateral)
	Putamen (Bilateral)	Precuneus (Bilateral)
	Rolandic_Oper (Bilateral)	Rectus (Bilateral)
	Supramarginal (Bilateral)	Rolandic_Oper (Bilateral)
	Supp_Motor_Area (Bilateral)	Supramarginal (Bilateral)
	Temporal_Inf (Bilateral)	Temporal_Inf (Bilateral)
	Temporal_Mid (Bilateral)	Temporal_Mid (Bilateral)
	Temporal_Pole_Mid (Bilateral)	Temporal_Pole_Mid (Bilateral)
	Temporal_Pole_Sup (Bilateral)	Temporal_Pole_Sup (Bilateral)
	Temporal_Sup (Bilateral)	Temporal_Sup (Bilateral) Vermis_4_5
GS	Amygdala_L	Angular (Bilateral)
	Calcarine_L	Calcarine (Bilateral)
	Cerebelum_Crus1_L	Cerebelum_6 (Bilateral)
	Cueneus (Bilateral)	Cerebelum_Crus1_L
	Frontal_Inf_Oper (Bilateral)	Cingulam_Mid_R
	Frontal_Inf_Tri (Bilateral)	Cingulam_Post_R
	Fusiform (Bilateral)	Cueneus (Bilateral)
	Hippocampus (Bilateral)	Fusiform (Bilateral)
	Insula_L	Heschl_R
	Lingual_R	Hippocampus_R
	Occipital_Inf_R	Insula (Bilateral)
	Occipital_Mid_L	Lingual (Bilateral)
	Occipital_Sup (Bilateral)	Occipital_Inf (Bilateral)
	Paracentral_Lobule_L	Occipital_Mid (Bilateral)
	ParaHippocampal_R	Occipital_Sup (Bilateral)
	Parietal_Inf (Bilateral)	ParaHippocampal (Bilateral)
	Parietal_Inf_L	Parietal_Inf (Bilateral)
	Parietal_Sup_R	Parietal_Sup (Bilateral)
	Postcentral (Bilateral)	Postcentral (Bilateral)
	Postcentral_L	Precentral_L
	Precentral_L	Precuneus (Bilateral)
	Precuneus (Bilateral)	Rolandic_Oper (Bilateral)
	Rolandic_Oper_L	Supp_Motor_Area_L
	Supramarginal (Bilateral)	SupraMarginal_L
	Temporal_Inf_R	Temporal_Mid (Bilateral)
	Temporal_Mid (Bilateral)	Temporal_Pole_Sup
	Temporal_Pole_Sup_L	(Bilateral)
	Temporal_Sup (Bilateral)	Temporal_Sup (Bilateral)
	Temporal_Sup_L	

**Fig. 2:**
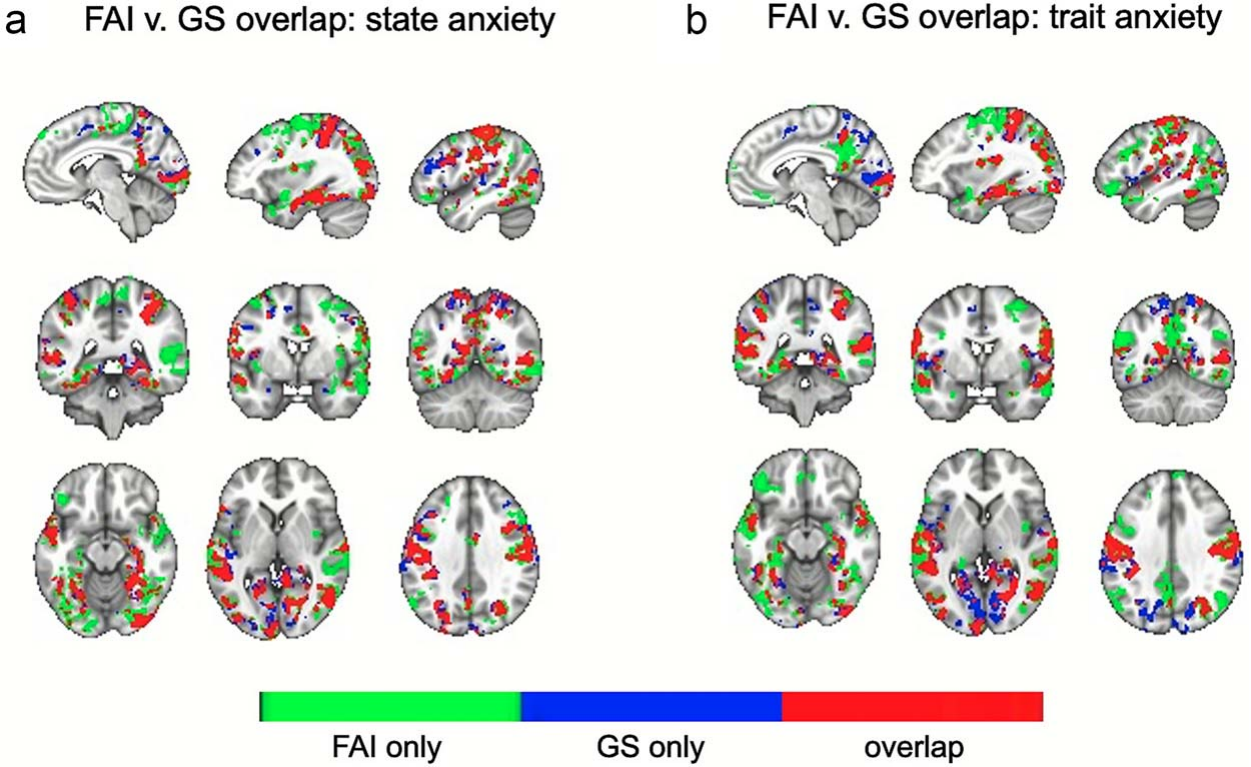
Spatial overlap in the relationship between global components and anxiety measures. For a) state anxiety and b) trait anxiety. Regions colored green are clusters where only FAI was associated with anxiety; regions colored in blue are clusters where only GS was associated with anxiety; those in red indicate areas of overlap.

The spatial overlap across maps relating FAI or GS to state or trait anxiety are depicted in Fig. 2. Areas of prominent overlap between FAI and GS for both state and trait anxiety included regions of parahippocampal gyrus and hippocampus, fusiform gyrus, superior parietal cortex, lateral occipital cortex, primary visual cortex, and anterior intraparietal sulcus. Regions in which the group-level FAI maps contained a larger number of significant voxels compared to GS maps for both anxiety measures included the left posterior insula, primary motor cortex, and right temporoparietal occipital junction. For both state and trait anxiety, a larger number of significant voxels were observed in the visual cortex for GS compared with FAI. These observations are based on the overlap maps shown in Fig. 2; we did not statistically test for differences in the magnitude of FAI versus GS effects at a given voxel.

### Temporal associations between global components and anxiety measures

**Fig. 3:**
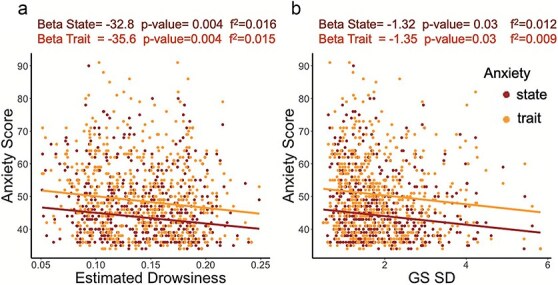
Relating temporal characteristics of global components with anxiety measures. a) Relationship between estimated drowsiness (standard deviation of FAI temporal signal) and anxiety. b) Relationship between standard deviation of the GS and anxiety.

The estimated drowsiness level (standard deviation of the FAI time-series) exhibited a significant negative relationship with both state and trait anxiety (β = −32.8, *P* = 0.004, f^2^ = 0.016; β = −35.6, *P* = 0.004, f^2^ = 0.015; Fig. 3). The standard deviation of the GS also had a negative relationship to state and trait anxiety, albeit with a weaker effect size (β = −1.32, *P* = 0.03, f^2^ = 0.012; β = −1.35, *P* = 0.03, f^2^ = 0.009). There were no significant associations between mean HR and state or trait anxiety measures (β = 0.05, *P* = 0.44, f^2^ = 0.002; β = −0.02, *P* = 0.77, f^2^ = 0.0003) (Supplementary Fig. S6a). We then examined effects of age, finding that both estimated drowsiness (r = −0.23, *P* < 0.001) and the standard deviation of the GS (r = −0.39, *P* = < 0.001) were significantly correlated with age. However, age did not relate to either state (r = 0.06, *P* = 0.14) or trait (r = 0.02, *P* = 0.61) anxiety measures, suggesting it does not drive the relationships between global fMRI effects and anxiety. Nonetheless, as a secondary check, we replicated the analysis after removing subjects older than 55 years. We found that the strength of associations diminished, although the direction of effects was preserved (Supplementary Fig. S7). Additionally, when we used the original sample of participants across the full age range but removed the age covariate, we found that the associations remained significant (and with similar effect sizes) across both trait and state anxiety for FAI (β = −34.8, *P* = 0.005, f^2^ = 0.014; β = −32.8, *P* = 0.005, f^2^ = 0.017) and GS (β = −1.2, *P* = 0.03, f^2^ = 0.008; β = −1.4, *P* = 0.01, f^2^ = 0.016). Further, adding mean FD as a group-level covariate (and maintaining the age covariate) strengthened the associations with both trait and state anxiety for FAI (β = −35.8, *P* = 0.002, f^2^ = 0.020; β = −37.8, *P* = 0.002, f^2^ = 0.017, respectively) and GS (β = −1.5, *P* = 0.009, f^2^ = 0.014; β = −1.7, *P* = 0.009, f^2^ = 0.013, respectively).

### Spatial overlap between resting-state networks and the association between global components and anxiety measures

**Fig. 4:**
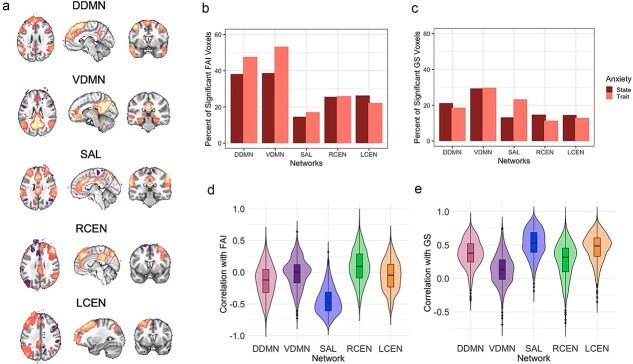
Alignment of resting-state networks with associations between global components and anxiety. a) Resting-state networks of interest, derived using ICA. b and c) Percentage of spatial overlap between resting-state networks of interest and maps associating global components to anxiety. d and e) Correlations between the time courses of the FAI or GS and the time courses of the indicated networks.

In the spatial patterns of association between global components and anxiety measures (Fig. 4), significant clusters in the FAI map overlapped most prominently with both subnetworks of the DMN, particularly for trait anxiety (Fig. 4b). Significant clusters in the GS map overlapped most strongly with VDMN. For the GS (Fig. 4c), trait and state anxiety diverged the most for SAL, which showed stronger overlap with trait anxiety.

### Relationship between global component signals and network signals

Correlations between the time-series of global components and functional networks are depicted in Fig. 4d and e. A repeated-measures ANOVA was conducted to examine whether global components differ in their correlation to the selected functional networks. There was a significant main effect of global component (F(2, 478) = 377, *P* < 0.001, η^2^ = 0.61), indicating that the correlations with networks differed across global components. Additionally, there was a significant global component × network interaction (F(8, 1912) = 144.1, *P* < 0.001, η^2^ = 0.38), suggesting that the effect of global component varied across networks. Follow-up pairwise comparisons were conducted using linear mixed-effects models with a random intercept for subject to allow estimation of standard errors and degrees of freedom. P-values were corrected for multiple comparisons using FDR at q = 0.05 and reported in Table 3. We also tested if the distribution of correlations between each network and each global component were significantly different from zero (Supplementary Table S1). All were significant except for the correlation between HR and VDMN.

**Table 3:** **a.** Pairwise contrasts the strength of association between each network and the indicated pair of global components: fMRI arousal index (fai), global signal (gs), heart rate (hr). P-values (q.value) are FDR-corrected for multiple comparisons.

Contrast	Network	Estimate	Standard Error	z.ratio	q.value
fai—gs	ddmn	−0.22	0.02	−13.42	<0.001
fai—hr	ddmn	0.00	0.02	−0.23	0.82
gs—hr	ddmn	0.22	0.02	13.19	<0.001
fai—gs	lcen	−0.40	0.02	−23.69	<0.001
fai—hr	lcen	−0.15	0.02	−9.25	<0.001
gs—hr	lcen	0.24	0.02	14.45	<0.001
fai—gs	rcen	−0.35	0.02	−20.84	<0.001
fai—hr	rcen	−0.28	0.02	−16.71	<.001
gs—hr	rcen	0.07	0.02	4.12	<0.001
fai—gs	sal	−0.08	0.02	−5.04	<0.001
fai—hr	sal	0.22	0.02	13.25	<0.001
gs—hr	sal	0.31	0.02	18.30	<0.001
fai—gs	vdmn	−0.11	0.02	−6.62	<0.001
fai—hr	vdmn	−0.16	0.02	−9.40	<0.001
gs—hr	vdmn	−0.05	0.02	−2.78	0.01

**Table 3:** **b.** Pairwise contrasts the strength of association within each network and the indicated pair of global components. P-values (q.value) are FDR-corrected for multiple comparisons.

Contrast	Global Component	Estimate	Standard Error	z.ratio	q.value
ddmn—lcen	fai	0.10	0.02	5.93	<0.001
ddmn—rcen	fai	0.24	0.02	14.27	<0.001
ddmn—sal	fai	−0.28	0.02	−16.89	<0.001
ddmn—vdmn	fai	0.14	0.02	8.40	<0.001
lcen—rcen	fai	0.14	0.02	8.34	<0.001
lcen—sal	fai	−0.38	0.02	−22.82	<0.001
lcen—vdmn	fai	0.04	0.02	2.47	0.01
rcen—sal	fai	−0.52	0.02	−31.16	<0.001
rcen—vdmn	fai	−0.10	0.02	−5.87	<0.001
sal—vdmn	fai	0.42	0.02	25.29	<0.001
ddmn—lcen	gs	−0.07	0.02	−4.34	<0.001
ddmn—rcen	gs	0.11	0.02	6.85	<0.001
ddmn—sal	gs	−0.14	0.02	−8.51	<0.001
ddmn—vdmn	gs	0.25	0.02	15.20	<0.001
lcen—rcen	gs	0.19	0.02	11.20	<0.001
lcen—sal	gs	−0.07	0.02	−4.17	<0.001
lcen—vdmn	gs	0.33	0.02	19.55	<0.001
rcen—sal	gs	−0.26	0.02	−15.37	<0.001
rcen—vdmn	gs	0.14	0.02	8.35	<0.001
sal—vdmn	gs	0.40	0.02	23.72	<0.001
ddmn—lcen	hr	−0.05	0.02	−3.09	0.01
ddmn—rcen	hr	−0.04	0.02	−2.21	0.05
ddmn—sal	hr	−0.06	0.02	−3.41	0.01
ddmn—vdmn	hr	−0.01	0.02	−0.77	0.49
lcen—rcen	hr	0.01	0.02	0.87	0.48
lcen—sal	hr	−0.01	0.02	−0.32	0.75
lcen—vdmn	hr	0.04	0.02	2.31	0.05
rcen—sal	hr	−0.02	0.02	−1.19	0.33
rcen—vdmn	hr	0.02	0.02	1.44	0.25
sal—vdmn	hr	0.04	0.02	2.64	0.03

### Impact of regressing global components on the association between functional connectivity and anxiety measures

The impact of regressing out global components on the association between network connectivity and anxiety measures is reported in Supplementary Tables S2a, S2b and S2c. Overall, we found no significant association between network connectivity and anxiety that survived correction for multiple comparisons (Supplementary Tables S2a,b), and that global component regression did not have a statistically significant impact on the association between functional connectivity and anxiety measures (Supplementary Table S2c).

## Discussion

In a large community sample of adults, we found that global fMRI components linked with cortical arousal were significantly related to both state and trait anxiety measures. These findings suggest that global fMRI effects may be a source of information—and a novel avenue for identifying biomarkers of anxiety—as opposed to a confound (Li et al. 2019a), and that careful consideration should be taken when determining whether to remove them in preprocessing.

We first tested if the brain-wide spatial distribution of the global mean signal (GS), an fMRI cortical arousal index (FAI), and heart rate variation would relate to inter-individual differences in state and trait anxiety. We observed that anxiety measures were linked with significant clusters within maps associated with the GS and FAI, but not heart rate. As shown in Figs. 2 and 3, regions passing the significance threshold in all maps were widely distributed across areas including the right hippocampus, fusiform, middle temporal gyrus, occipital cortex, calcarine, lingual gyrus, and intraparietal sulcus. To our knowledge, this study is the first to investigate which regions strongly associate cortical arousal to anxiety. From previous rodent studies investigating the etiology of anxiety, three of these regions had been noted to play a pivotal role in processing affective components of anxiety: the hippocampus processes information that corresponds with fearful or aversive events (Hill et al. 2021; Bernier et al. 2017); the fusiform gyrus is found to show increased activity when presented with emotional stimuli to promote saliency (Edmiston et al. 2024); and the medial temporal lobe stores memories related to the anxious experience (Dolcos et al. 2004). Our findings suggest that the expression of cortical arousal in the human brain is correlated with state and trait anxiety within brain regions that are critical to the emotional processing of anxiety.

Secondly, we investigated whether the temporal characteristics of these global components were related to anxiety. We computed the standard deviation of the FAI and GS, which estimate an individual’s level of drowsiness, (Wong et al. 2013; Falahpour et al. 2018) and found that as estimated drowsiness decreased (indicating higher arousal levels), both state and trait anxiety significantly increased. These findings support theories suggesting that higher arousal is associated with greater anxiety (Noteboom et al. 2001). This represents a meaningful contribution to the field, as it indicates that the temporal features of fMRI global components are sensitive to arousal-related characteristics linked with anxiety. These findings also emphasize that global components often considered artifactual may, in fact, contain valuable information related to anxiety.

In relating network signals with global component time-courses, the salience network emerged as exhibiting the most prominent relationships. First, the time-series of the salience network had the closest negative relationship with FAI and strongest positive relationship with GS. These findings support the possibility that arousal may be crucial in the activation of the salience network at rest and potentially during cognitive tasks (Unsworth and Robison 2017; Robison and Brewer 2020; Tripathi et al. 2025). Although the salience network showed a different pattern of correlation with heart rate compared to FAI and GS, direct comparison across these measures is limited because heart rate correlations were estimated using a lagged cross-correlation approach, whereas FAI and GS correlations were computed using a single (zero-lag) correlation. The close relationship between the salience network and the FAI may also be anticipated from the fact that the salience network exhibits the strongest negative amplitudes within the fMRI arousal template (Supplementary Fig. S8).

State and trait anxiety measures tend to be positively associated (Li and Jiang 2022; De la Peña-Arteaga et al. 2024), and were found to have a correlation of r = 0.75 in our sample. This observation accounts for the general similarity between state and trait anxiety in their spatial and temporal associations with fMRI global components. Nonetheless, some qualitative distinctions are apparent as well. For example, FAI exhibits significant group-level clusters across a larger portion of the default-mode network in its association with trait anxiety (Figs. 1, 2, 4b). Further study of distinctions between state and trait anxiety in the context of global fMRI components would be a valuable future direction.

Given the wide age range of the present sample, we additionally examined whether age may impact the key results (specifically, the spatial and temporal relationship between global components and anxiety measures). When constraining the age range to 18 to 55 years, fewer regions survived statistical thresholding in the spatial associations between global components and anxiety. Those which survived in both the full-age-range (Fig. 1) and this restricted age range (Supplementary Fig. S5) included temporal and occipital regions*.* One potential reason for the weaker effects within the younger age group involves the smaller sample size of the 18 to 55-year group (*n* = 324, versus *n* = 543 for the full group). Another factor could involve a potential interaction between arousal effects and age. Indeed, the arousal system is known to decline with age (Mak and Schneider 2021), and it is possible that our analyses may be detecting age-related changes in the relationship between arousal circuits and anxiety, which may be an interesting avenue for future investigation. In addition, age-related anatomic changes may pose a confound. Despite careful attention to registration during preprocessing, and controlling for age in our analyses, this possibility cannot be fully ruled out. Future studies examining how fMRI correlates of arousal relate to anxiety in different age groups could help to further disentangle these effects.

Several factors are important to consider in interpreting the present results. First, STAI measures were taken one day after the fMRI scan; to our knowledge, the exact time difference between these measurements is not provided with the data and therefore could not be controlled in our study. Second, data were drawn from a community sample rather than from a clinical population, which could potentially explain our observation that the relationship between network connectivity and anxiety levels was weaker and more variable than in certain patient studies. Next, the FAI reflects an estimated contribution of cortical arousal to fMRI signals. Simultaneous EEG or pupillometry and fMRI may more directly determine the extent to which cortical arousal effects relate to anxiety and/or contribute to network connectivity in anxiety disorders. In addition, fMRI measures and anxiety scores were examined only at the cross-subject level. Future studies may want to examine within-subject relationships to examine how cortical arousal manifests within the same subjects while they experience different anxiety levels. Finally, our findings may be limited to the resting-state experimental environment. The interplay between HRV and brain networks may be more dynamic during real-world conditions (Chand 2020, Unsworth and Robison 2017; Robison and Brewer 2020; Tripathi et al. 2025), which may also explain why heart rate did not relate to anxiety in the present study.

Head motion is also an important consideration in this study, given the evidence that head motion is related to both global fMRI components and anxiety (Power et al. 2017; Gu et al. 2022). Here, we mitigated effects of head motion at the subject level through FIX ICA processing, and further tested whether the significant findings would be impacted by inclusion of a group-level mean FD covariate. Interestingly, including mean FD increased the strength of spatial and temporal association between global components and anxiety (Supplementary Fig. S3), suggesting that the information linking cortical arousal and anxiety arises from neurophysiological effects and not solely driven by head motion.

To conclude, we find that global effects in fMRI, including those linked with cortical arousal, are closely tied with anxiety measures. These findings support the emerging viewpoint that global components—which are typically regarded as confounds in fMRI studies—may hold valuable information related to anxiety and perhaps to a broader range of clinical questions. We encourage future investigation into arousal measures in fMRI studies as potential predictive markers or within the context of mechanistic models.

## Supplementary Material

NKI_ANX_GLOBAL_MANUSCRIPT_ACCEPTED_FIN_SUPPLEMENTARY_MATERIAL_bhag008

## Data Availability

This study used data from the Enhanced Nathan Kline Institute—Rockland Sample. The neuroimaging data from this sample is publicly available, and phenotypic data are available with a Data Use Agreement from the NKI. The website for accessing the data is https://fcon_1000.projects.nitrc.org/indi/enhanced/. Code for reproducing this study will be available on github.com/neurdylab/globalfMRI_anx_proj and preliminary code is currently available on github.com/krogge-obando/globalfMRI_anx_proj/.
