# Supplementary material for: Global cortical arousal effects in fMRI reveal brain markers of state and trait anxiety
Source: Cereb Cortex. 2026 Feb 17;36(2):bhag008. doi: 10.1093/cercor/bhag008 (PMC13370124; doi:10.1093/cercor/bhag008)
Supplement: NKI_ANX_GLOBAL_MANUSCRIPT_ACCEPTED_FIN_SUPPLEMENTARY_MATERIAL_bhag008 [file nki_anx_global_manuscript_accepted_fin_supplementary_material_bhag008.docx]

**Global cortical arousal effects in fMRI reveal brain markers of state and trait anxiety**

Kimberly Kundert-Obando^1,2*^, Terra Lee^3,4,7†^, Caroline G. Martin^3,10†^, Kamalpreet Kaur^10^, Juan Gomez Lagandara^10^ , Yamin Li^9^, Jeffrey M. Harding^10^, Shiyu Wang^6^, Richard Song^7,9^, Ruoqi Yang^8,9^, Rithwik Guntaka^10^, Sarah E. Goodale^5,6^, Roza G. Bayrak^10^, Lucina Q. Uddin^11,12^, Martin Walter^13^, Jeremy Hogeveen^14^, Catie Chang^2,6,9,10*^

1. Neuroscience Graduate Program, Vanderbilt University, Nashville, TN, USA
2. Vanderbilt Brain Institute, Vanderbilt University, Nashville, TN, USA
3. Vanderbilt University Medical Center, Nashville, TN, USA
4. Department of Psychology and Human Development, Vanderbilt University, Nashville, TN, USA
5. [Vanderbilt Memory and Alzheimer's Center](https://www.vumc.org/vmac/), Vanderbilt University Medical Center, Nashville, TN, USA
6. Department of Biomedical Engineering, Vanderbilt University, Nashville, TN, USA
7. Program in Neuroscience, Vanderbilt University, Nashville, TN, USA
8. Department of Computer Science and Engineering, University of California, San Diego, CA, USA
9. Department of Computer Science, Vanderbilt University, Nashville, TN, USA
10. Department of Electrical and Computer Engineering, Vanderbilt University, Nashville, TN, USA
11. Department of Psychiatry and Biobehavioral Sciences, University of California Los Angeles, Los Angeles, CA, USA
12. Department of Psychology, University of California Los Angeles, Los Angeles, CA, USA
13. University Clinic for Psychiatry and Psychotherapy, University Hospital Jena, Jena, Germany
14. Department of Psychology, University of New Mexico, NM, USA

^†^ equal contribution

***Correspondence:**

Kimberly Kundert-Obando (recent name change), [kimberly.k.rogge-obando@vanderbilt.edu](mailto:kimberly.k.rogge-obando@vanderbilt.edu)

Catie Chang, [catie.chang@vanderbilt.edu](mailto:catie.chang@vanderbilt.edu)

400 24th Avenue S

Nashville, TN 37240

**
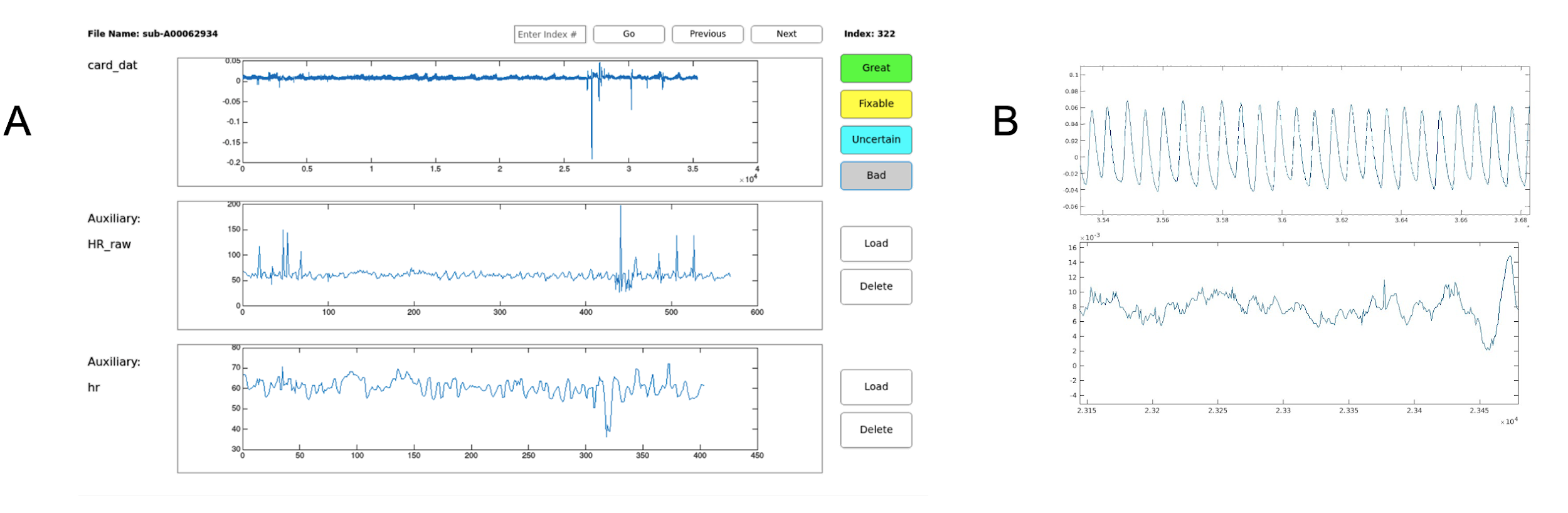
**

**Supplementary Figure 1.** **Visual inspection of physiological data quality.** The user interface for manual inspection of PPG data. We inspected three signals: raw PPG data (card_dat), an instantaneous heart rate measure (inter-beat-interval series, converted to HR by taking the inverse and multiplying by 60; HR_raw) and lastly, a heart rate fMRI regressor sampled at each TR (hr). B) An example of high- (top) vs. low- (bottom) quality PPG data.


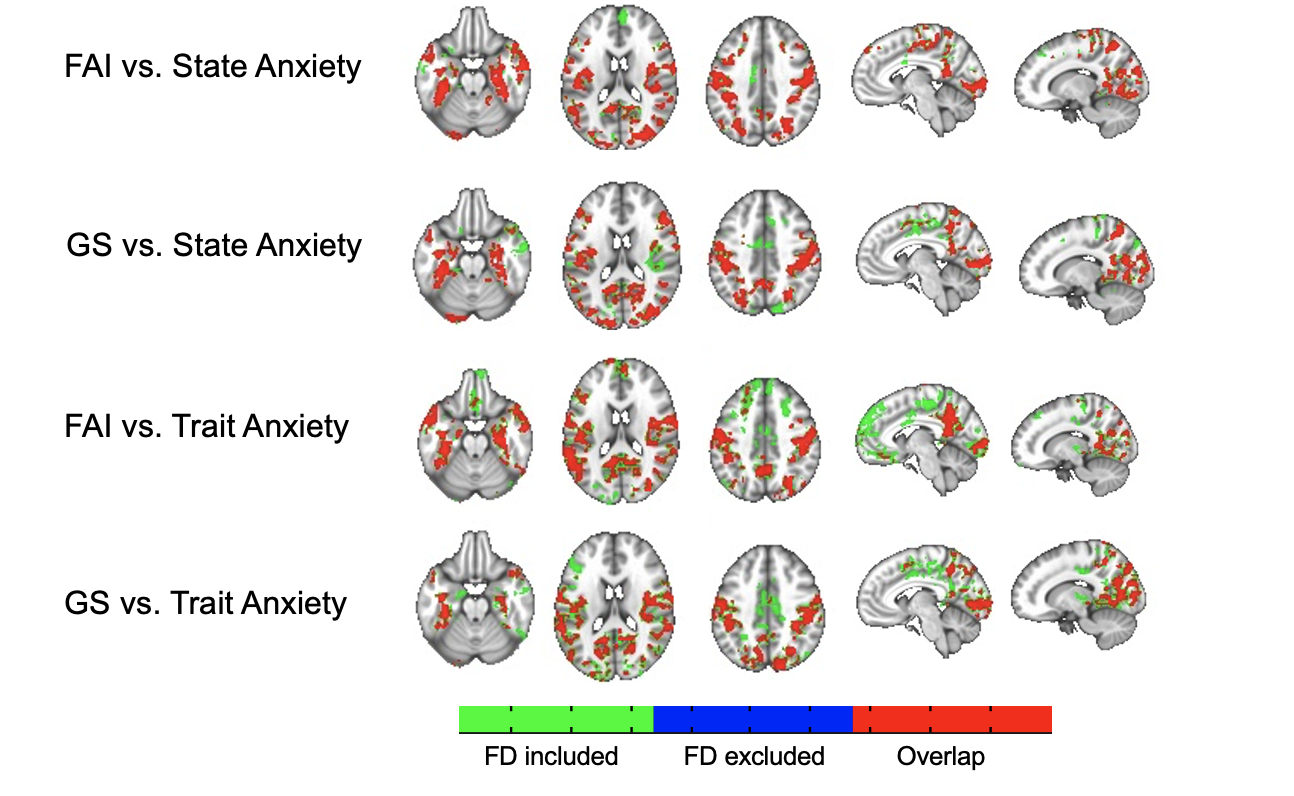


**Supplementary Figure 2.** Effect of covarying for head motion (mean framewise displacement; FD) on the spatial association between global components and anxiety.

**
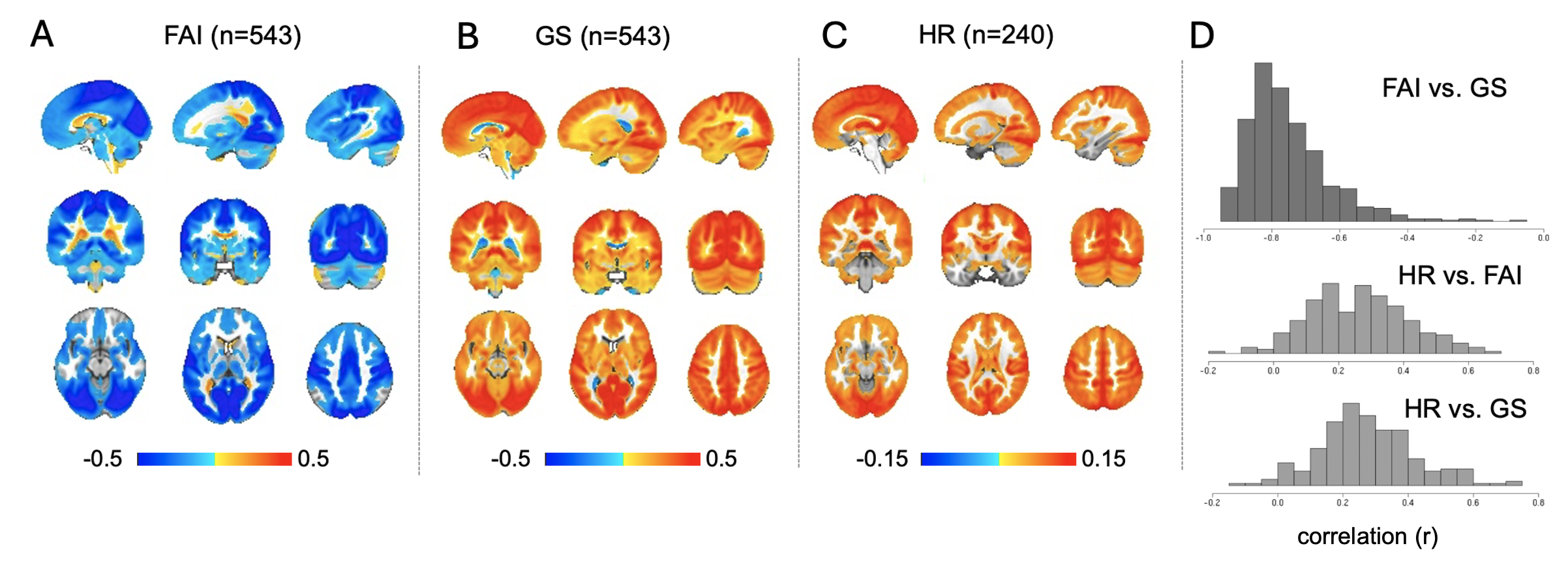
**

**Supplementary Figure 3. Global spatial and temporal characteristics of global components.** For the data used in this study, maps depict spatial patterns averaged across the indicated samples corresponding to A) the fMRI Arousal Index (FAI), B) the global mean signal (GS), and C) heart rate (HR). D) Temporal correlations between global components**.**


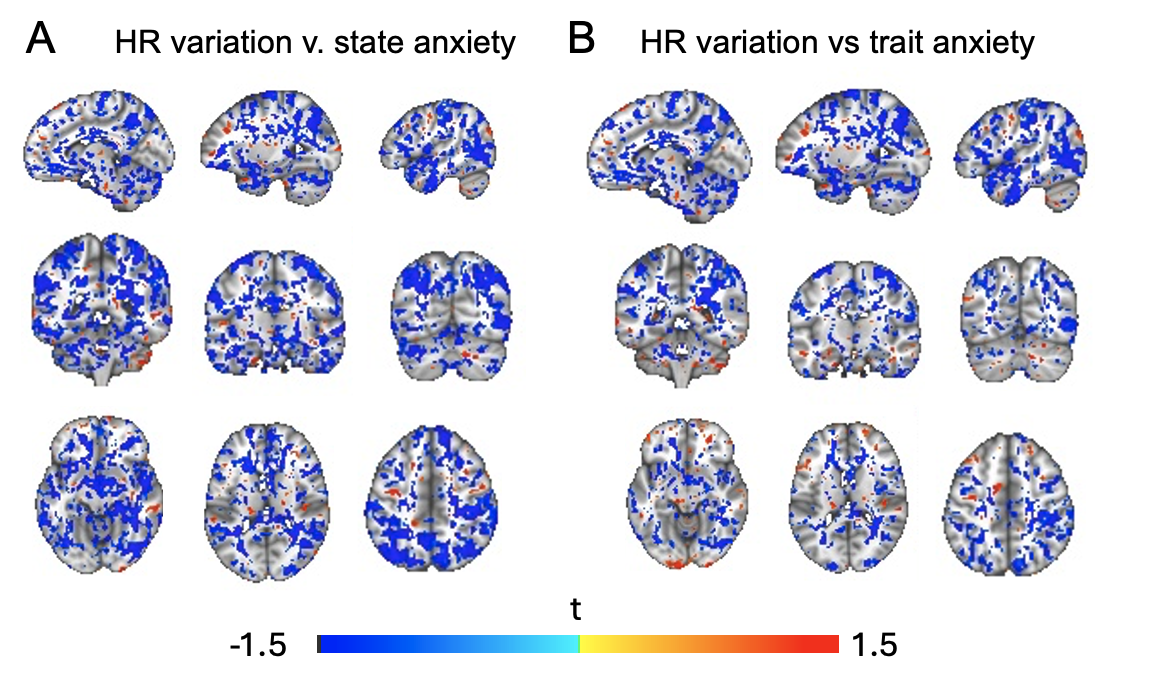


**Supplementary Figure 4.** Non-significant associations between percent variance explained by heart rate with A) state and B) trait anxiety measures.


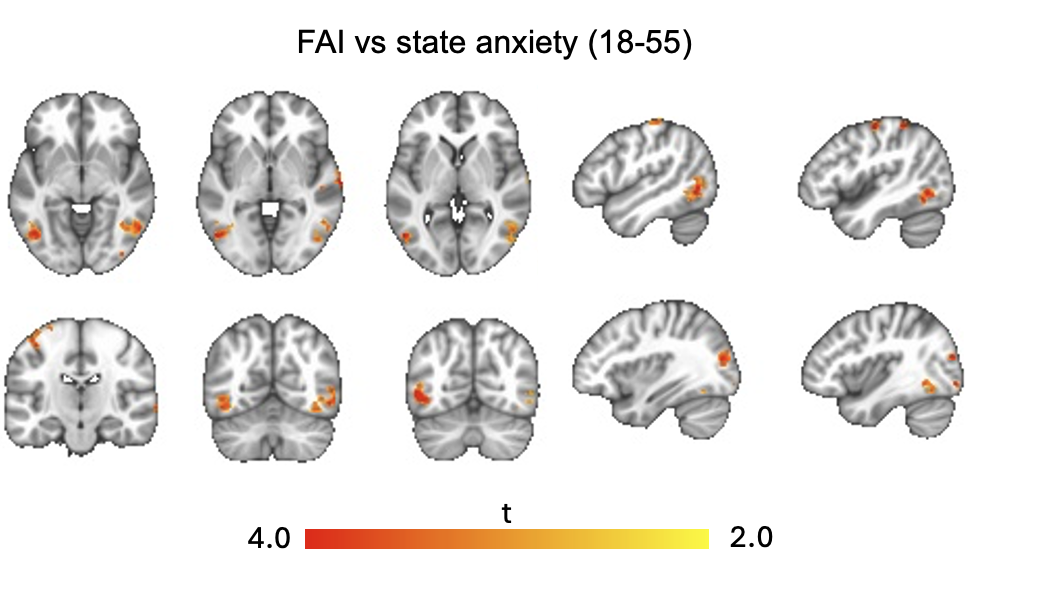


**Supplementary Figure 5.** Association between FAI and state anxiety in a subset of the data with participant ages between 18-55 years.

**
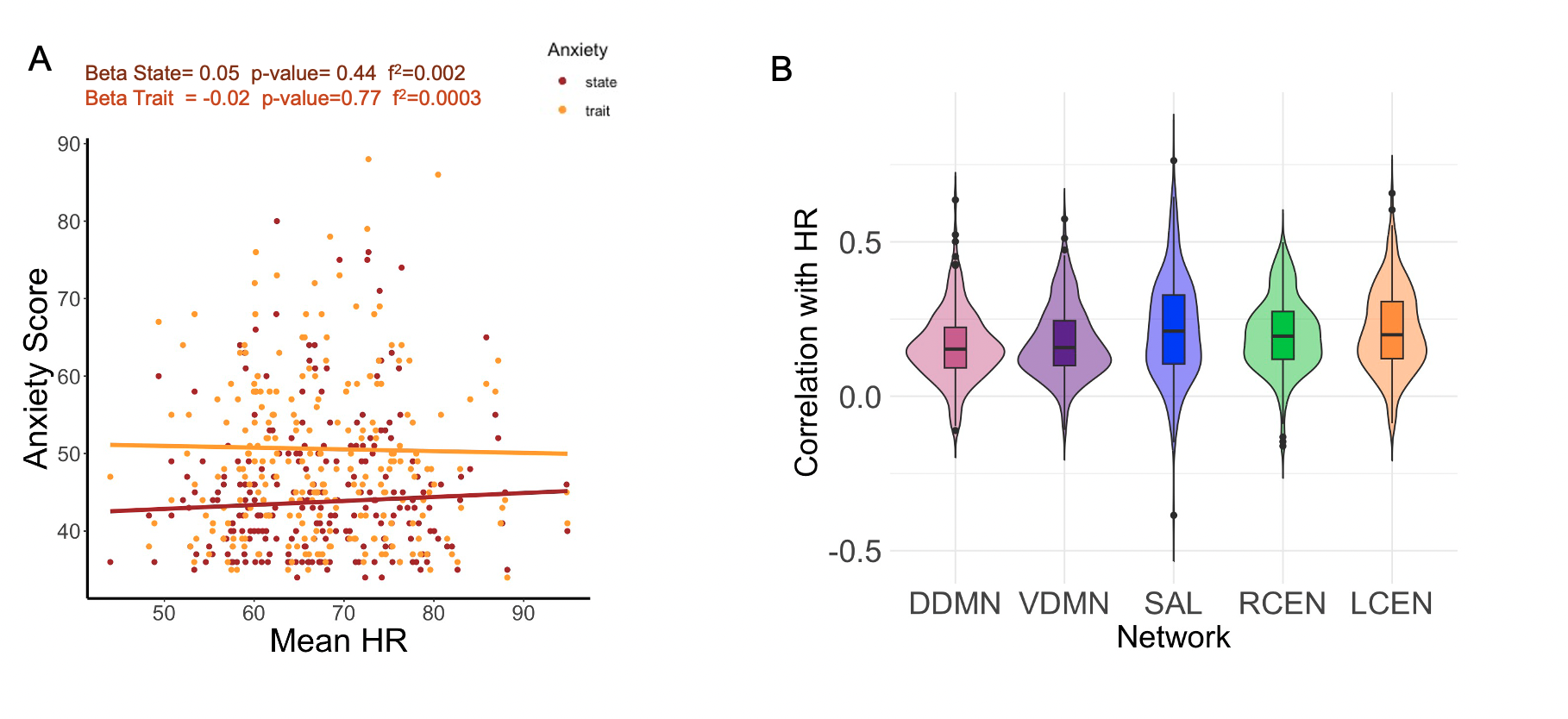
**

**Supplementary Figure 6.**  A) Correlation between mean HR and state and trait anxiety scores. B) Maximum cross correlation between HR and fMRI network time courses.

**
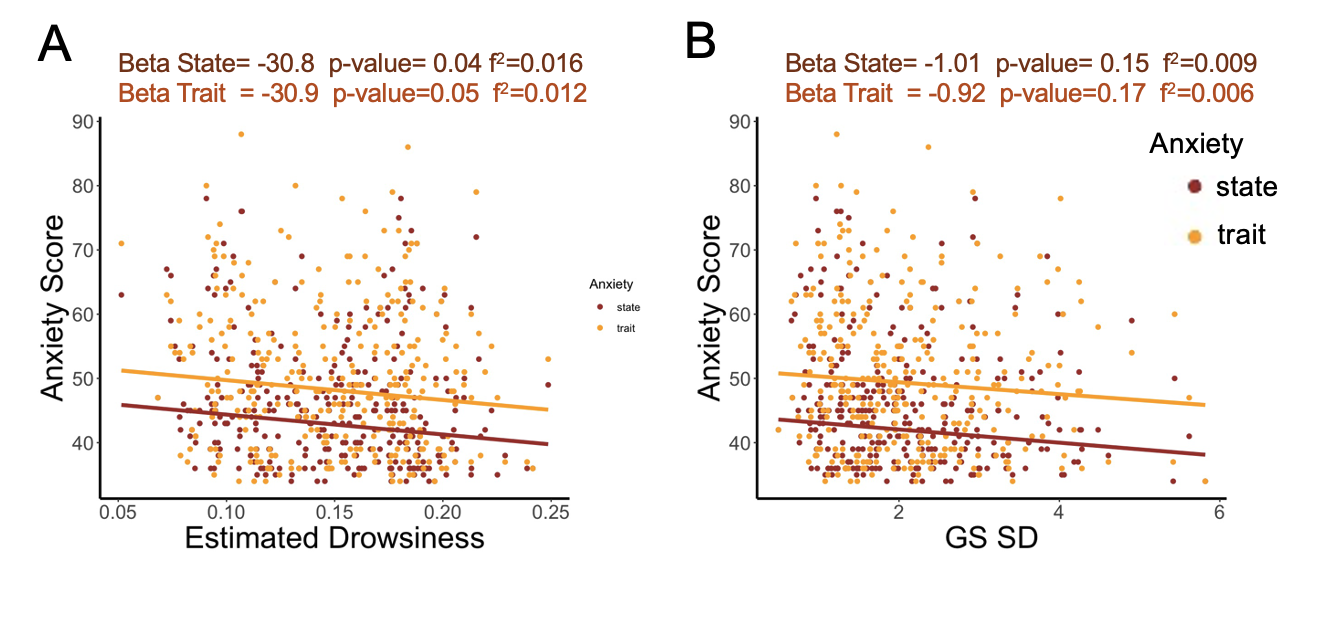
**

**Supplementary Figure 7.** Temporal associations between global components and anxiety in the subset of participants aged 18-55.

**
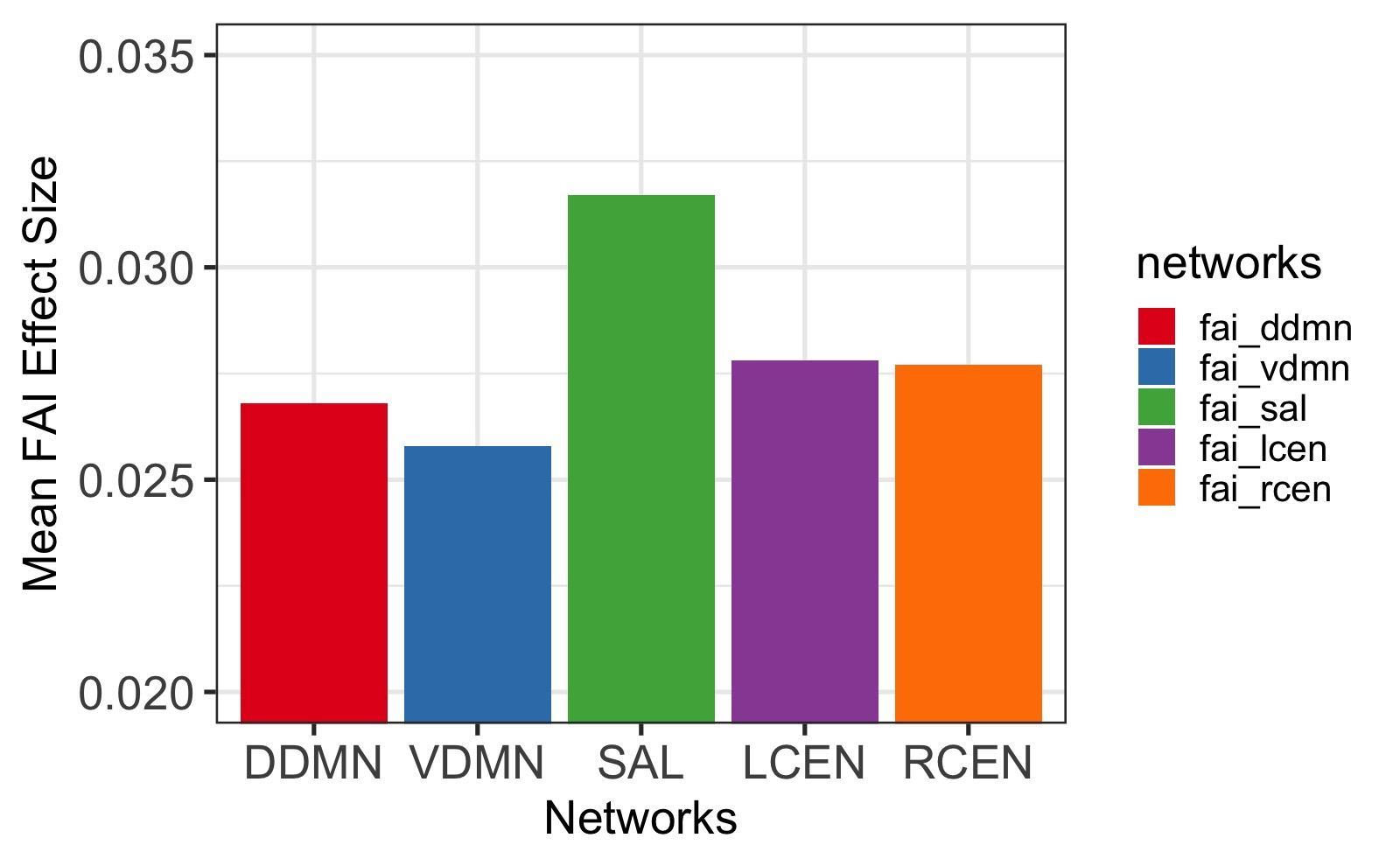
**

**Supplementary Figure 8.** Values of the fMRI arousal template (Goodale et al., 2021) averaged within regions corresponding to each of the networks of interest.

**Supplementary Table 1. Testing whether temporal correlations between functional networks and global components are significantly different from zero.**

|  | ***t-*statistic** | **BH FDRs** | **Adjusted p-values (q)** | **Raw p-values** |
| --- | --- | --- | --- | --- |
| G_ddmn.t | 42.46 | 1.51E-59 | <0.001 | <0.001 |
| G_vdmn.t | 13.95 | 5.83E-64 | <0.001 | <0.001 |
| G_sal.t | 58.79 | 7.08E-55 | <0.001 | <0.001 |
| G_rcen.t | 24.27 | 1.23E-72 | <0.001 | <0.001 |
| G_lcen.t | 51.84 | 2.79E-67 | <0.001 | <0.001 |
| A_ddmn.t | -12.50 | 5.97E-174 | <0.001 | <0.001 |
| A_vdmn.t | -1.65 | 7.21E-38 | <0.001 | <0.001 |
| A_sal.t | -48.71 | 3.42E-236 | <0.001 | <0.001 |
| A_rcen.t | 8.44 | 3.70E-88 | <0.001 | <0.001 |
| A_lcen.t | -5.29 | 2.51E-211 | <0.001 | <0.001 |
| H_ddmn.t | 22.12 | 1.37E-31 | <0.001 | <0.001 |
| H_vdmn.t | 23.55 | 0.10 | 0.10 | 0.10 |
| H_sal.t | 20.65 | 1.87E-199 | <0.001 | <0.001 |
| H_rcen.t | 26.48 | 3.35E-16 | <0.001 | <0.001 |
| H_lcen.t | 24.66 | 1.95E-07 | <0.001 | <0.001 |

X_net.t corresponds to a one sample t-test of the correlation between network “net” and global component “X”. G: global signal, A: FAI and H: heart rate.

| **Supplementary Table 2a. Impact of regressing out global components on the relationship between network FC and state or trait anxiety (543 subjects).**   \| **Regression** \| **Network FC** \| **state_Beta** \| **state_pval** \| **state_eff** \| **state_age_eff** \| \| --- \| --- \| --- \| --- \| --- \| --- \| \| **Non Regressed** \| **ddmn.vdmn** \| -2.67E-04 \| 7.39E-01 \| 2.07E-04 \| 5.89E-02 \| \| **ddmn.sal** \| -7.43E-04 \| 5.06E-01 \| 8.23E-04 \| 7.16E-03 \| \| **ddmn.lcen** \| -7.62E-04 \| 3.66E-01 \| 1.52E-03 \| 1.05E-05 \| \| **ddmn.rcen** \| -2.75E-04 \| 7.56E-01 \| 1.80E-04 \| 4.57E-04 \| \| **vdmn.sal** \| -2.43E-03 \| 2.34E-02 \| 9.60E-03 \| 1.85E-04 \| \| **vdmn.rcen** \| -5.30E-04 \| 5.52E-01 \| 6.57E-04 \| 9.89E-03 \| \| **vdmn.lcen** \| -1.83E-04 \| 8.34E-01 \| 8.19E-05 \| 3.52E-02 \| \| **lcen.sal** \| -3.54E-04 \| 7.50E-01 \| 1.89E-04 \| 1.65E-02 \| \| **lcen.rcen** \| -1.51E-04 \| 8.53E-01 \| 6.36E-05 \| 7.00E-08 \| \| **rcen.sal** \| -2.04E-03 \| 3.99E-02 \| 7.89E-03 \| 3.82E-03 \| \|  \|  \|  \|  \|  \|  \| \| **FAI Regressed** \| **ddmn.vdmn** \| -3.63E-04 \| 6.52E-01 \| 3.78E-04 \| 6.12E-02 \| \| **ddmn.sal** \| -6.35E-04 \| 5.15E-01 \| 7.89E-04 \| 1.00E-03 \| \| **ddmn.lcen** \| -1.04E-03 \| 1.96E-01 \| 3.11E-03 \| 1.97E-05 \| \| **ddmn.rcen** \| -1.97E-04 \| 8.19E-01 \| 9.74E-05 \| 2.43E-04 \| \| **vdmn.sal** \| -2.27E-03 \| 1.79E-02 \| 1.05E-02 \| 5.08E-04 \| \| **vdmn.rcen** \| -5.04E-04 \| 5.74E-01 \| 5.90E-04 \| 1.11E-02 \| \| **vdmn.lcen** \| -1.79E-04 \| 8.35E-01 \| 8.09E-05 \| 4.69E-02 \| \| **lcen.sal** \| -2.25E-04 \| 8.41E-01 \| 7.51E-05 \| 4.55E-03 \| \| **lcen.rcen** \| 4.68E-05 \| 9.54E-01 \| 6.20E-06 \| 2.47E-04 \| \| **rcen.sal** \| -1.98E-03 \| 5.10E-02 \| 7.11E-03 \| 7.69E-03 \| \|  \|  \|  \|  \|  \|  \| \| **GS Regressed** \| **ddmn.vdmn** \| 1.49E-04 \| 8.55E-01 \| 6.23E-05 \| 3.61E-02 \| \| **ddmn.sal** \| -1.06E-03 \| 2.38E-01 \| 2.59E-03 \| 3.66E-03 \| \| **ddmn.lcen** \| -1.15E-03 \| 1.79E-01 \| 3.36E-03 \| 3.91E-03 \| \| **ddmn.rcen** \| -3.32E-04 \| 7.16E-01 \| 2.46E-04 \| 1.26E-03 \| \| **vdmn.sal** \| -1.89E-03 \| 3.63E-02 \| 8.18E-03 \| 3.80E-03 \| \| **vdmn.rcen** \| -1.01E-04 \| 9.09E-01 \| 2.42E-05 \| 4.51E-05 \| \| **vdmn.lcen** \| -2.97E-05 \| 9.73E-01 \| 2.19E-06 \| 2.76E-02 \| \| **lcen.sal** \| -3.27E-04 \| 7.64E-01 \| 1.67E-04 \| 3.20E-03 \| \| **lcen.rcen** \| -6.31E-04 \| 5.14E-01 \| 7.92E-04 \| 2.39E-03 \| \| **rcen.sal** \| -2.18E-03 \| 3.54E-02 \| 8.27E-03 \| 1.14E-02 \|            \| **Regression** \| **Network FC** \| **trait_Beta** \| **trait_pval** \| **trait_eff** \| **trait_age_eff** \| \| --- \| --- \| --- \| --- \| --- \| --- \| \| **Non Regressed** \| **ddmn.vdmn** \| -2.26E-04 \| 7.47E-01 \| 1.93E-04 \| 5.87E-02 \| \| **ddmn.sal** \| -9.44E-04 \| 3.35E-01 \| 1.73E-03 \| 7.37E-03 \| \| **ddmn.lcen** \| -1.19E-05 \| 9.87E-01 \| 4.85E-07 \| 2.75E-05 \| \| **ddmn.rcen** \| 9.89E-04 \| 2.03E-01 \| 3.02E-03 \| 5.13E-04 \| \| **vdmn.sal** \| -1.92E-03 \| 4.19E-02 \| 7.73E-03 \| 8.87E-05 \| \| **vdmn.rcen** \| -1.13E-04 \| 8.82E-01 \| 4.07E-05 \| 3.51E-02 \| \| **vdmn.lcen** \| 4.18E-04 \| 5.93E-01 \| 5.31E-04 \| 9.61E-03 \| \| **lcen.sal** \| -5.71E-04 \| 5.58E-01 \| 6.39E-04 \| 1.66E-02 \| \| **lcen.rcen** \| -7.80E-05 \| 9.13E-01 \| 2.22E-05 \| 3.96E-07 \| \| **rcen.sal** \| -2.19E-03 \| 1.20E-02 \| 1.18E-02 \| 3.42E-03 \| \|  \|  \|  \|  \|  \|  \| \| **FAI Regressed** \| **ddmn.vdmn** \| -2.00E-04 \| 7.77E-01 \| 1.50E-04 \| 6.09E-02 \| \| **ddmn.sal** \| -3.56E-04 \| 6.77E-01 \| 3.22E-04 \| 9.27E-04 \| \| **ddmn.lcen** \| -1.40E-04 \| 8.43E-01 \| 7.32E-05 \| 5.21E-05 \| \| **ddmn.rcen** \| 1.17E-03 \| 1.20E-01 \| 4.51E-03 \| 2.81E-04 \| \| **vdmn.sal** \| -1.37E-03 \| 1.04E-01 \| 4.94E-03 \| 3.22E-04 \| \| **vdmn.rcen** \| -2.24E-04 \| 7.66E-01 \| 1.65E-04 \| 4.69E-02 \| \| **vdmn.lcen** \| 4.98E-04 \| 5.26E-01 \| 7.47E-04 \| 1.09E-02 \| \| **lcen.sal** \| -5.30E-04 \| 5.90E-01 \| 5.40E-04 \| 4.59E-03 \| \| **lcen.rcen** \| 4.24E-06 \| 9.95E-01 \| 6.62E-08 \| 2.51E-04 \| \| **rcen.sal** \| -2.28E-03 \| 1.03E-02 \| 1.23E-02 \| 7.18E-03 \| \|  \|  \|  \|  \|  \|  \| \| **GS Regressed** \| **ddmn.vdmn** \| -6.70E-05 \| 9.25E-01 \| 1.63E-05 \| 3.64E-02 \| \| **ddmn.sal** \| -5.87E-04 \| 4.57E-01 \| 1.03E-03 \| 3.39E-03 \| \| **ddmn.lcen** \| -1.45E-04 \| 8.47E-01 \| 6.90E-05 \| 3.56E-03 \| \| **ddmn.rcen** \| 9.78E-04 \| 2.21E-01 \| 2.79E-03 \| 1.18E-03 \| \| **vdmn.sal** \| -9.36E-04 \| 2.38E-01 \| 2.60E-03 \| 4.32E-03 \| \| **vdmn.rcen** \| -1.90E-04 \| 8.03E-01 \| 1.16E-04 \| 2.77E-02 \| \| **vdmn.lcen** \| 7.87E-04 \| 3.11E-01 \| 1.91E-03 \| 3.67E-05 \| \| **lcen.sal** \| -2.69E-04 \| 7.79E-01 \| 1.46E-04 \| 3.28E-03 \| \| **lcen.rcen** \| -8.75E-04 \| 3.02E-01 \| 1.98E-03 \| 2.30E-03 \| \| **rcen.sal** \| -2.28E-03 \| 1.17E-02 \| 1.19E-02 \| 1.07E-02 \|   **Supplementary Table 2b. Impact of regressing out global components on the relationship between network FC and state or trait anxiety (240 subjects).**   \| **Regression** \| **Network FC** \| **state_Beta** \| **state_pval** \| **state_eff** \| **state_age_eff** \| \| --- \| --- \| --- \| --- \| --- \| --- \| \| **Non Regressed** \| **ddmn.vdmn** \| 9.41E-04 \| 4.68E-01 \| 2.25E-03 \| 6.49E-02 \| \| **ddmn.sal** \| -1.19E-03 \| 5.09E-01 \| 1.86E-03 \| 4.72E-03 \| \| **ddmn.lcen** \| 2.81E-04 \| 8.38E-01 \| 1.79E-04 \| 5.63E-04 \| \| **ddmn.rcen** \| 1.29E-03 \| 3.86E-01 \| 3.21E-03 \| 5.75E-05 \| \| **vdmn.sal** \| -7.13E-04 \| 6.95E-01 \| 6.55E-04 \| 8.31E-04 \| \| **vdmn.rcen** \| -1.42E-03 \| 3.27E-01 \| 4.10E-03 \| 3.97E-03 \| \| **vdmn.lcen** \| -2.14E-04 \| 8.82E-01 \| 9.43E-05 \| 2.67E-02 \| \| **lcen.sal** \| 9.73E-04 \| 5.96E-01 \| 1.20E-03 \| 1.21E-02 \| \| **lcen.rcen** \| 7.81E-04 \| 5.54E-01 \| 1.50E-03 \| 6.03E-03 \| \| **rcen.sal** \| -2.03E-03 \| 2.20E-01 \| 6.43E-03 \| 9.61E-03 \| \|  \|  \|  \|  \|  \|  \| \| **HR regressed** \| **ddmn.vdmn** \| 8.62E-04 \| 4.92E-01 \| 2.01E-03 \| 5.68E-02 \| \| **ddmn.sal** \| -1.49E-03 \| 3.79E-01 \| 3.31E-03 \| 5.36E-03 \| \| **ddmn.lcen** \| 3.21E-04 \| 8.12E-01 \| 2.42E-04 \| 2.48E-04 \| \| **ddmn.rcen** \| 1.42E-03 \| 3.42E-01 \| 3.85E-03 \| 1.01E-03 \| \| **vdmn.sal** \| -9.58E-04 \| 5.51E-01 \| 1.51E-03 \| 2.10E-06 \| \| **vdmn.rcen** \| -1.53E-03 \| 2.63E-01 \| 5.35E-03 \| 7.10E-04 \| \| **vdmn.lcen** \| 1.18E-05 \| 9.93E-01 \| 2.90E-07 \| 2.02E-02 \| \| **lcen.sal** \| 7.73E-04 \| 6.60E-01 \| 8.28E-04 \| 1.24E-02 \| \| **lcen.rcen** \| 5.64E-04 \| 6.60E-01 \| 8.27E-04 \| 4.88E-03 \| \| **rcen.sal** \| -2.14E-03 \| 1.71E-01 \| 8.02E-03 \| 7.64E-03 \| \|  \|  \|  \|  \|  \|  \| \| **FAI regressed** \| **ddmn.vdmn** \| 9.04E-04 \| 4.75E-01 \| 2.17E-03 \| 6.71E-02 \| \| **ddmn.sal** \| -7.74E-04 \| 6.21E-01 \| 1.04E-03 \| 1.95E-03 \| \| **ddmn.lcen** \| -1.87E-04 \| 8.81E-01 \| 9.50E-05 \| 6.36E-05 \| \| **ddmn.rcen** \| 1.48E-03 \| 3.01E-01 \| 4.58E-03 \| 8.40E-05 \| \| **vdmn.sal** \| -1.03E-03 \| 5.19E-01 \| 1.78E-03 \| 7.34E-04 \| \| **vdmn.rcen** \| -1.14E-03 \| 4.21E-01 \| 2.77E-03 \| 5.87E-03 \| \| **vdmn.lcen** \| -1.00E-04 \| 9.43E-01 \| 2.15E-05 \| 3.42E-02 \| \| **lcen.sal** \| 1.52E-03 \| 4.03E-01 \| 2.99E-03 \| 7.34E-03 \| \| **lcen.rcen** \| 5.12E-04 \| 6.95E-01 \| 6.55E-04 \| 6.68E-03 \| \| **rcen.sal** \| -1.39E-03 \| 4.11E-01 \| 2.89E-03 \| 1.07E-02 \| \|  \|  \|  \|  \|  \|  \| \| **GS regressed** \| **ddmn.vdmn** \| 1.05E-03 \| 4.25E-01 \| 2.72E-03 \| 3.61E-02 \| \| **ddmn.sal** \| -1.82E-03 \| 2.26E-01 \| 6.27E-03 \| 2.55E-03 \| \| **ddmn.lcen** \| -2.28E-04 \| 8.68E-01 \| 1.18E-04 \| 2.00E-03 \| \| **ddmn.rcen** \| 1.80E-03 \| 2.38E-01 \| 5.96E-03 \| 5.84E-04 \| \| **vdmn.sal** \| -1.41E-03 \| 3.56E-01 \| 3.63E-03 \| 3.40E-03 \| \| **vdmn.rcen** \| -1.28E-03 \| 3.64E-01 \| 3.51E-03 \| 3.54E-03 \| \| **vdmn.lcen** \| -5.81E-04 \| 6.92E-01 \| 6.71E-04 \| 1.36E-02 \| \| **lcen.sal** \| 7.45E-04 \| 6.83E-01 \| 7.11E-04 \| 5.82E-03 \| \| **lcen.rcen** \| 3.60E-04 \| 8.17E-01 \| 2.28E-04 \| 4.57E-03 \| \| **rcen.sal** \| -2.18E-03 \| 2.24E-01 \| 6.32E-03 \| 1.32E-02 \|      \| **Regression** \| **Network FC** \| **trait_Beta** \| **trait_pval** \| **trait_eff** \| **trait_age_eff** \| \| --- \| --- \| --- \| --- \| --- \| --- \| \| **Non Regressed** \| **ddmn.vdmn** \| 2.05E-04 \| 8.55E-01 \| 1.42E-04 \| 6.60E-02 \| \| **ddmn.sal** \| -3.75E-04 \| 8.10E-01 \| 2.47E-04 \| 5.04E-03 \| \| **ddmn.lcen** \| 4.85E-04 \| 6.83E-01 \| 7.13E-04 \| 6.51E-04 \| \| **ddmn.rcen** \| 2.73E-03 \| 3.33E-02 \| 1.95E-02 \| 1.57E-06 \| \| **vdmn.sal** \| 3.13E-04 \| 8.42E-01 \| 1.69E-04 \| 8.08E-04 \| \| **vdmn.rcen** \| -1.20E-03 \| 3.35E-01 \| 3.98E-03 \| 2.57E-02 \| \| **vdmn.lcen** \| -1.77E-05 \| 9.89E-01 \| 8.40E-07 \| 3.64E-03 \| \| **lcen.sal** \| 2.99E-03 \| 5.91E-02 \| 1.53E-02 \| 1.07E-02 \| \| **lcen.rcen** \| 1.38E-03 \| 2.26E-01 \| 6.28E-03 \| 6.90E-03 \| \| **rcen.sal** \| -2.02E-03 \| 1.60E-01 \| 8.46E-03 \| 8.21E-03 \| \|  \|  \|  \|  \|  \|  \| \| **HR regressed** \| **ddmn.vdmn** \| 8.95E-05 \| 9.34E-01 \| 2.89E-05 \| 5.76E-02 \| \| **ddmn.sal** \| -5.32E-04 \| 7.17E-01 \| 5.59E-04 \| 5.85E-03 \| \| **ddmn.lcen** \| 6.16E-04 \| 5.98E-01 \| 1.19E-03 \| 3.25E-04 \| \| **ddmn.rcen** \| 2.74E-03 \| 3.36E-02 \| 1.95E-02 \| 5.26E-04 \| \| **vdmn.sal** \| -2.54E-05 \| 9.85E-01 \| 1.42E-06 \| 9.07E-06 \| \| **vdmn.rcen** \| -1.22E-03 \| 3.21E-01 \| 4.20E-03 \| 1.94E-02 \| \| **vdmn.lcen** \| -2.45E-04 \| 8.37E-01 \| 1.82E-04 \| 5.33E-04 \| \| **lcen.sal** \| 2.72E-03 \| 7.24E-02 \| 1.39E-02 \| 1.11E-02 \| \| **lcen.rcen** \| 9.79E-04 \| 3.77E-01 \| 3.34E-03 \| 5.44E-03 \| \| **rcen.sal** \| -1.84E-03 \| 1.75E-01 \| 7.88E-03 \| 6.35E-03 \| \|  \|  \|  \|  \|  \|  \| \| **FAI regressed** \| **ddmn.vdmn** \| 1.31E-04 \| 9.05E-01 \| 6.10E-05 \| 6.81E-02 \| \| **ddmn.sal** \| -4.75E-04 \| 7.26E-01 \| 5.25E-04 \| 1.75E-03 \| \| **ddmn.lcen** \| 3.82E-04 \| 7.25E-01 \| 5.28E-04 \| 5.26E-05 \| \| **ddmn.rcen** \| 2.62E-03 \| 3.38E-02 \| 1.94E-02 \| 5.15E-09 \| \| **vdmn.sal** \| 3.56E-04 \| 7.96E-01 \| 2.85E-04 \| 6.89E-04 \| \| **vdmn.rcen** \| -1.16E-03 \| 3.44E-01 \| 3.83E-03 \| 3.31E-02 \| \| **vdmn.lcen** \| 4.07E-04 \| 7.41E-01 \| 4.66E-04 \| 5.70E-03 \| \| **lcen.sal** \| 2.29E-03 \| 1.46E-01 \| 9.04E-03 \| 6.28E-03 \| \| **lcen.rcen** \| 1.04E-03 \| 3.58E-01 \| 3.61E-03 \| 7.35E-03 \| \| **rcen.sal** \| -2.38E-03 \| 1.03E-01 \| 1.14E-02 \| 9.33E-03 \| \|  \|  \|  \|  \|  \|  \| \| **GS regressed** \| **ddmn.vdmn** \| -1.54E-04 \| 8.92E-01 \| 7.82E-05 \| 3.66E-02 \| \| **ddmn.sal** \| -1.10E-03 \| 3.97E-01 \| 3.06E-03 \| 1.99E-03 \| \| **ddmn.lcen** \| -2.11E-04 \| 8.59E-01 \| 1.35E-04 \| 1.91E-03 \| \| **ddmn.rcen** \| 2.44E-03 \| 6.40E-02 \| 1.47E-02 \| 1.09E-03 \| \| **vdmn.sal** \| 3.27E-04 \| 8.05E-01 \| 2.61E-04 \| 3.57E-03 \| \| **vdmn.rcen** \| -1.59E-03 \| 2.08E-01 \| 6.77E-03 \| 1.25E-02 \| \| **vdmn.lcen** \| 3.84E-04 \| 7.52E-01 \| 4.24E-04 \| 3.68E-03 \| \| **lcen.sal** \| 2.44E-03 \| 1.22E-01 \| 1.03E-02 \| 5.00E-03 \| \| **lcen.rcen** \| 3.65E-05 \| 9.78E-01 \| 3.12E-06 \| 4.65E-03 \| \| **rcen.sal** \| -2.69E-03 \| 8.27E-02 \| 1.29E-02 \| 1.14E-02 \|   The notation “x.y” denotes functional connectivity between networks x and y. The rightmost column shows the effect size of age within the model. Abbreviations: dorsal default mode network (ddmn), ventral default mode network (vdmn), salience network (sal), left central executive network (lcen), and right central executive network(rcen), partial effect size f^2^ (eff)  **Supplementary Table 2c. Interaction test of the impact of pre and post global regression association between network functional connectivity and anxiety.**  **State Anxiety** | | | | | | |
| --- | --- | --- | --- | --- | --- | --- | --- | --- | --- | --- | --- | --- | --- | --- | --- | --- | --- | --- | --- | --- | --- | --- | --- | --- | --- | --- | --- | --- | --- | --- | --- | --- | --- | --- | --- | --- | --- | --- | --- | --- | --- | --- | --- | --- | --- | --- | --- | --- | --- | --- | --- | --- | --- | --- | --- | --- | --- | --- | --- | --- | --- | --- | --- | --- | --- | --- | --- | --- | --- | --- | --- | --- | --- | --- | --- | --- | --- | --- | --- | --- | --- | --- | --- | --- | --- | --- | --- | --- | --- | --- | --- | --- | --- | --- | --- | --- | --- | --- | --- | --- | --- | --- | --- | --- | --- | --- | --- | --- | --- | --- | --- | --- | --- | --- | --- | --- | --- | --- | --- | --- | --- | --- | --- | --- | --- | --- | --- | --- | --- | --- | --- | --- | --- | --- | --- | --- | --- | --- | --- | --- | --- | --- | --- | --- | --- | --- | --- | --- | --- | --- | --- | --- | --- | --- | --- | --- | --- | --- | --- | --- | --- | --- | --- | --- | --- | --- | --- | --- | --- | --- | --- | --- | --- | --- | --- | --- | --- | --- | --- | --- | --- | --- | --- | --- | --- | --- | --- | --- | --- | --- | --- | --- | --- | --- | --- | --- | --- | --- | --- | --- | --- | --- | --- | --- | --- | --- | --- | --- | --- | --- | --- | --- | --- | --- | --- | --- | --- | --- | --- | --- | --- | --- | --- | --- | --- | --- | --- | --- | --- | --- | --- | --- | --- | --- | --- | --- | --- | --- | --- | --- | --- | --- | --- | --- | --- | --- | --- | --- | --- | --- | --- | --- | --- | --- | --- | --- | --- | --- | --- | --- | --- | --- | --- | --- | --- | --- | --- | --- | --- | --- | --- | --- | --- | --- | --- | --- | --- | --- | --- | --- | --- | --- | --- | --- | --- | --- | --- | --- | --- | --- | --- | --- | --- | --- | --- | --- | --- | --- | --- | --- | --- | --- | --- | --- | --- | --- | --- | --- | --- | --- | --- | --- | --- | --- | --- | --- | --- | --- | --- | --- | --- | --- | --- | --- | --- | --- | --- | --- | --- | --- | --- | --- | --- | --- | --- | --- | --- | --- | --- | --- | --- | --- | --- | --- | --- | --- | --- | --- | --- | --- | --- | --- | --- | --- | --- | --- | --- | --- | --- | --- | --- | --- | --- | --- | --- | --- | --- | --- | --- | --- | --- | --- | --- | --- | --- | --- | --- | --- | --- | --- | --- | --- | --- | --- | --- | --- | --- | --- | --- | --- | --- | --- | --- | --- | --- | --- | --- | --- | --- | --- | --- | --- | --- | --- | --- | --- | --- | --- | --- | --- | --- | --- | --- | --- | --- | --- | --- | --- | --- | --- | --- | --- | --- | --- | --- | --- | --- | --- | --- | --- | --- | --- | --- | --- | --- | --- | --- | --- | --- | --- | --- | --- | --- | --- | --- | --- | --- | --- | --- | --- | --- | --- | --- | --- | --- | --- | --- | --- | --- | --- | --- | --- | --- | --- | --- | --- | --- | --- | --- | --- | --- | --- | --- | --- | --- | --- | --- | --- | --- | --- | --- | --- | --- | --- | --- | --- | --- | --- | --- | --- | --- | --- | --- | --- | --- | --- | --- | --- | --- | --- | --- | --- | --- | --- | --- | --- | --- | --- | --- | --- | --- | --- | --- | --- | --- | --- | --- | --- | --- | --- | --- | --- | --- | --- | --- | --- | --- | --- | --- | --- | --- | --- | --- | --- | --- | --- | --- | --- | --- | --- | --- | --- | --- | --- | --- | --- | --- | --- | --- | --- | --- | --- | --- | --- | --- | --- | --- | --- | --- | --- | --- | --- | --- | --- | --- | --- | --- | --- | --- | --- | --- | --- | --- | --- | --- | --- | --- | --- | --- | --- | --- | --- | --- | --- | --- | --- | --- | --- | --- | --- | --- | --- | --- | --- | --- | --- | --- | --- | --- | --- | --- | --- | --- | --- | --- | --- | --- | --- | --- | --- | --- | --- | --- | --- | --- | --- | --- | --- | --- | --- | --- | --- | --- | --- | --- | --- | --- | --- | --- | --- | --- | --- | --- | --- | --- | --- | --- | --- | --- | --- | --- | --- | --- | --- | --- | --- | --- | --- | --- | --- | --- | --- | --- | --- | --- | --- | --- | --- | --- | --- | --- | --- | --- | --- | --- | --- | --- | --- | --- | --- | --- | --- | --- | --- | --- | --- | --- | --- | --- | --- | --- | --- | --- | --- | --- | --- | --- | --- | --- | --- | --- | --- | --- | --- | --- | --- | --- | --- | --- | --- | --- | --- | --- | --- | --- | --- | --- | --- | --- | --- | --- | --- | --- | --- | --- | --- | --- | --- | --- | --- | --- | --- | --- | --- | --- | --- | --- | --- | --- | --- | --- | --- | --- | --- | --- | --- | --- | --- | --- | --- | --- | --- | --- | --- | --- | --- | --- | --- | --- | --- | --- | --- | --- | --- | --- | --- | --- | --- | --- | --- | --- | --- | --- | --- | --- | --- | --- | --- | --- | --- | --- | --- | --- | --- | --- | --- | --- | --- | --- | --- | --- | --- | --- | --- | --- | --- | --- | --- | --- | --- | --- | --- | --- | --- | --- | --- | --- | --- | --- | --- | --- | --- | --- | --- |
| **pre-post FAI** | **f2.Marginal R2** | **Estimate** | **Std. Error** | **t-value** | **p-value** | **q-pval** |
| ddmn.vdmn | 3.65E-06 | 7.31E-05 | 2.94E-04 | 0.25 | 0.80 | 0.99 |
| ddmn.sal | 1.09E-04 | -5.17E-04 | 5.61E-04 | -0.92 | 0.36 | 0.99 |
| ddmn.lcen | 1.24E-04 | 4.26E-04 | 3.26E-04 | 1.31 | 0.19 | 0.99 |
| ddmn.rcen | 4.85E-06 | -8.94E-05 | 2.75E-04 | -0.32 | 0.75 | 0.99 |
| vdmn.sal | 2.31E-06 | -7.38E-05 | 4.74E-04 | -0.16 | 0.88 | 0.99 |
| vdmn.lcen | 6.72E-07 | -3.42E-05 | 3.17E-04 | -0.11 | 0.91 | 0.99 |
| vdmn.rcen | 4.43E-08 | -8.86E-06 | 2.61E-04 | -0.03 | 0.97 | 0.99 |
| lcen.sal | 6.05E-07 | -4.16E-05 | 5.49E-04 | -0.08 | 0.94 | 0.99 |
| lcen.rcen | 4.69E-05 | -2.62E-04 | 2.13E-04 | -1.23 | 0.22 | 0.99 |
| **pre-post GS** | **f2.Marginal R2** | **Estimate** | **Std. Error** | **t-value** | **p-value** | **q-pval** |
| ddmn.vdmn | 9.29E-05 | -3.72E-04 | 4.41E-04 | -0.85 | 0.40 | 0.99 |
| ddmn.sal | 3.19E-05 | -2.71E-04 | 8.14E-04 | -0.33 | 0.74 | 0.99 |
| ddmn.lcen | 7.79E-05 | 3.52E-04 | 4.40E-04 | 0.80 | 0.42 | 0.99 |
| ddmn.rcen | 4.29E-05 | -2.75E-04 | 6.22E-04 | -0.44 | 0.66 | 0.99 |
| vdmn.sal | 2.45E-05 | -2.34E-04 | 6.76E-04 | -0.35 | 0.73 | 0.99 |
| vdmn.lcen | 4.37E-06 | -8.80E-05 | 3.59E-04 | -0.25 | 0.81 | 0.99 |
| vdmn.rcen | 2.13E-05 | -1.94E-04 | 5.26E-04 | -0.37 | 0.71 | 0.99 |
| lcen.sal | 2.52E-07 | -2.68E-05 | 8.17E-04 | -0.03 | 0.97 | 0.99 |
| lcen.rcen | 3.73E-05 | 2.59E-04 | 5.71E-04 | 0.45 | 0.65 | 0.99 |
| **pre-post HR** | **f2.Marginal R2** | **Estimate** | **Std. Error** | **t-value** | **p-value** | **q-pval** |
| ddmn.vdmn | 5.61E-05 | 3.03E-04 | 3.38E-04 | 0.89 | 0.37 | 0.99 |
| ddmn.sal | 2.37E-05 | 2.68E-04 | 5.02E-04 | 0.53 | 0.59 | 0.99 |
| ddmn.lcen | 1.79E-05 | 1.81E-04 | 3.61E-04 | 0.50 | 0.62 | 0.99 |
| ddmn.rcen | 8.68E-09 | 4.28E-06 | 4.55E-04 | 0.01 | 0.99 | 0.99 |
| vdmn.sal | 5.21E-05 | 3.78E-04 | 6.01E-04 | 0.63 | 0.53 | 0.99 |
| vdmn.lcen | 1.23E-05 | -1.59E-04 | 3.70E-04 | -0.43 | 0.67 | 0.99 |
| vdmn.rcen | 7.60E-05 | 3.75E-04 | 4.55E-04 | 0.83 | 0.41 | 0.99 |
| lcen.sal | 4.98E-05 | 4.07E-04 | 5.55E-04 | 0.73 | 0.46 | 0.99 |
| lcen.rcen | 2.96E-05 | 2.22E-04 | 3.98E-04 | 0.56 | 0.58 | 0.99 |

| **Trait Anxiety** | | | | | | |
| --- | --- | --- | --- | --- | --- | --- |
| **pre-post FAI** | **f2.Marginal R2** | **Estimate** | **Std. Error** | **t-value** | **p-value** | **q p-values** |
| ddmn.vdmn | 1.98E-05 | -1.50E-04 | 2.59E-04 | -0.58 | 0.56 | 0.97 |
| ddmn.sal | 2.48E-04 | -6.84E-04 | 4.94E-04 | -1.39 | 0.17 | 0.97 |
| ddmn.lcen | 4.65E-05 | 2.30E-04 | 2.88E-04 | 0.80 | 0.42 | 0.97 |
| ddmn.rcen | 1.47E-05 | -1.37E-04 | 2.42E-04 | -0.56 | 0.57 | 0.97 |
| vdmn.sal | 1.31E-04 | -4.91E-04 | 4.17E-04 | -1.18 | 0.24 | 0.97 |
| vdmn.lcen | 5.96E-06 | 8.98E-05 | 2.79E-04 | 0.32 | 0.75 | 0.97 |
| vdmn.rcen | 4.05E-06 | -7.47E-05 | 2.30E-04 | -0.33 | 0.75 | 0.97 |
| lcen.sal | 5.53E-07 | 3.51E-05 | 4.83E-04 | 0.07 | 0.94 | 0.97 |
| lcen.rcen | 1.22E-05 | -1.18E-04 | 1.87E-04 | -0.63 | 0.53 | 0.97 |
| **pre-post GS** | **f2.Marginal R2** | **Estimate** | **Std. Error** | **t value** | **p-value** | **q p-values** |
| ddmn.vdmn | 1.01E-04 | -3.41E-04 | 3.88E-04 | -0.88 | 0.38 | 0.97 |
| ddmn.sal | 1.52E-04 | -5.21E-04 | 7.17E-04 | -0.73 | 0.47 | 0.97 |
| ddmn.lcen | 4.32E-06 | 7.32E-05 | 3.88E-04 | 0.19 | 0.85 | 0.97 |
| ddmn.rcen | 9.77E-06 | -1.15E-04 | 5.48E-04 | -0.21 | 0.83 | 0.97 |
| vdmn.sal | 4.87E-04 | -9.18E-04 | 5.94E-04 | -1.54 | 0.12 | 0.97 |
| vdmn.lcen | 1.27E-07 | 1.32E-05 | 3.16E-04 | 0.04 | 0.97 | 0.97 |
| vdmn.rcen | 1.35E-04 | -4.31E-04 | 4.63E-04 | -0.93 | 0.35 | 0.97 |
| lcen.sal | 1.65E-05 | -1.91E-04 | 7.20E-04 | -0.27 | 0.79 | 0.97 |
| lcen.rcen | 3.69E-04 | 7.15E-04 | 5.02E-04 | 1.42 | 0.15 | 0.97 |
| **pre-post HR** | **f2.Marginal R2** | **Estimate** | **Std. Error** | **t value** | **p-value** | **q-values** |
| ddmn.vdmn | 4.43E-06 | 7.38E-05 | 2.93E-04 | 0.25 | 0.80 | 0.97 |
| ddmn.sal | 1.85E-06 | 6.50E-05 | 4.35E-04 | 0.15 | 0.88 | 0.97 |
| ddmn.lcen | 3.19E-06 | -6.62E-05 | 3.12E-04 | -0.21 | 0.83 | 0.97 |
| ddmn.rcen | 1.28E-06 | 4.47E-05 | 3.93E-04 | 0.11 | 0.91 | 0.97 |
| vdmn.sal | 5.12E-05 | 3.24E-04 | 5.20E-04 | 0.62 | 0.53 | 0.97 |
| vdmn.lcen | 2.53E-06 | -6.24E-05 | 3.20E-04 | -0.19 | 0.85 | 0.97 |
| vdmn.rcen | 7.95E-05 | 3.33E-04 | 3.94E-04 | 0.85 | 0.40 | 0.97 |
| lcen.sal | 3.54E-05 | 2.95E-04 | 4.80E-04 | 0.61 | 0.54 | 0.97 |
| lcen.rcen | 7.46E-05 | 3.04E-04 | 3.44E-04 | 0.88 | 0.38 | 0.97 |

The notation “x.y” denotes functional connectivity between networks x and y. The rightmost column shows the effect size of age within the model. Abbreviations: dorsal default mode network (ddmn), ventral default mode network (vdmn), salience network (sal), left central executive network (lcen), and right central executive network (rcen)
